# Mice with reduced expression of the telomere‐associated protein Ft1 develop p53‐sensitive progeroid traits

**DOI:** 10.1111/acel.12730

**Published:** 2018-04-10

**Authors:** Mattia La Torre, Chiara Merigliano, Romina Burla, Carla Mottini, Giorgia Zanetti, Simona Del Giudice, Mariateresa Carcuro, Ilaria Virdia, Elisabetta Bucciarelli, Isabella Manni, Gianluca Rampioni Vinciguerra, Giulia Piaggio, Mara Riminucci, Ana Cumano, Armando Bartolazzi, Fiammetta Vernì, Silvia Soddu, Maurizio Gatti, Isabella Saggio

**Affiliations:** ^1^ Dipartimento di Biologia e Biotecnologie “C. Darwin” Sapienza Università di Roma Rome Italy; ^2^ Dipartimento di Ricerca, Diagnostica Avanzata e Innovazione Tecnologica Istituto Nazionale Tumori Regina Elena Rome Italy; ^3^ Istituto di Biologia e Patologia Molecolari del CNR Rome Italy; ^4^ Azienda Ospedaliera Sant'Andrea Rome Italy; ^5^ Dipartimento di Medicina Molecolare Sapienza Università di Roma Rome Italy; ^6^ Lymphopoiesis Unit Institut Pasteur Paris France

**Keywords:** aging, AKTIP, DNA damage, lamins, progeria, telomeres

## Abstract

Human AKTIP and mouse Ft1 are orthologous ubiquitin E2 variant proteins involved in telomere maintenance and DNA replication. AKTIP also interacts with A‐ and B‐type lamins. These features suggest that Ft1 may be implicated in aging regulatory pathways. Here, we show that cells derived from hypomorph *Ft1* mutant (*Ft1* ^*kof/kof*^) mice exhibit telomeric defects and that *Ft1* ^*kof/kof*^ animals develop progeroid traits, including impaired growth, skeletal and skin defects, abnormal heart tissue, and sterility. We also demonstrate a genetic interaction between *Ft1* and *p53*. The analysis of mice carrying mutations in both *Ft1* and *p53* (*Ft1* ^*kof/kof*^ *; p53* ^*ko/ko*^ and *Ft1* ^*kof/kof*^ *; p53* ^*+/ko*^) showed that reduction in p53 rescues the progeroid traits of *Ft1* mutants, suggesting that they are at least in part caused by a p53‐dependent DNA damage response. Conversely, *Ft1* reduction alters lymphomagenesis in *p53* mutant mice. These results identify Ft1 as a new player in the aging process and open the way to the analysis of its interactions with other progeria genes using the mouse model.

## 1 INTRODUCTION

Human AKTIP, mouse Ft1, and *Drosophila* Pendolino (Peo) are orthologous ubiquitin E2 variant proteins involved in telomere maintenance (Burla et al., 2015; Cenci, Ciapponi et al., 2015). AKTIP mediates proper telomere replication, binds telomeric DNA and the shelterins TRF1 and TRF2, and interacts with the DNA replication machinery components PCNA and RPA70. We have previously suggested that AKTIP works in concert with TRF1 to facilitate telomeric DNA replication (Burla et al., 2015).

AKTIP also interacts with A‐ and B‐type lamins and is enriched at the nuclear rim (Burla, Carcuro, et al., 2016). Reduction in AKTIP in human fibroblasts results in senescent phenotypes, including the activation of the p53 pathway, nuclear deformity, heterochromatin alterations, and senescence. In addition, AKTIP reduction affects lamin A expression in human cells (Burla, Carcuro, et al., 2016). Altogether, the properties of AKTIP place this protein at the crossroad of multiple pathways that have been associated with progeroid phenotypes.

The Hutchinson–Gilford progeria syndrome (HGPS) is the best‐characterized example of progeria, caused by a mutation in exon 11 of the *LMNA* gene leading to the production of a truncated form of lamin A (De Sandre‐Giovannoli et al., 2003). Patients with HGPS develop multiorgan abnormalities, including skeletal defects and absence of subcutaneous fat. They show a limited growth and die in the teenage years, prevalently due to cardiovascular problems leading to infarction or stroke (Hennekam, 2006). Mouse models reflect several aspects of the human disease; the LAKI model, for example, carrying the G608G mutation in the *LMNA* gene, is characterized by reduced lifespan and body weight, and skeletal and skin defects (Osorio, Navarro, et al., 2011).

The idea that lamins play a pivotal role in determining premature aging is also supported by the discovery of progeroid disorders different from HGPS. For example, restrictive dermopathy patients carry recessive mutations in the *ZMPSTE24* gene, which encodes the proteolytic enzyme involved in lamin A maturation (Barrowman, Wiley, Hudon‐Miller, Hrycyna & Michaelis, 2012). Also, in this case, a mouse model replicates the progeroid phenotype of the disease (Osorio, Ugalde, et al., 2011). A partial recovery of the *ZMPSTE24* ^*−/−*^ phenotype is obtained by depletion of the tumor suppressor protein p53, pointing to a role of DNA damage in the pathophysiology of this progeria (Varela et al., 2005).

In addition to the *LMNA* gene, several genes involved in DNA metabolism have been implicated in progeroid syndromes. They include the *WRN* and *BLM* genes, which encode members of the RecQ DNA helicase family and are responsible for the Werner and Bloom syndromes, respectively (Bachrati & Hickson, 2003).

Telomere dysfunctions have also been linked to progerias. Dyskeratosis congenita, which is caused by mutations in telomere‐related genes, has progeroid phenotypic traits (Dokal, 2011).

The involvement of AKTIP in telomere maintenance and regulation of lamin A (Burla et al., 2015; Burla, Carcuro, et al., 2016) prompted us to investigate whether this protein contributes to preventing premature aging. We thus generated mice bearing a mutation in the *Ft1* gene. We report here that *Ft1* mutant mice exhibit multiple progeroid traits, including impaired growth, skeletal and skin defects, and sterility. We also demonstrate an interplay between *Ft1* and *p53*. *Ft1* mutant mice carrying mutations in *p53* (*Ft1* ^*kof/kof*^ *;p53* ^*ko/ko*^ and *Ft1* ^*kof/kof*^ *;p53* ^*+/ko*^) showed a partial rescue of the progeroid traits observed in *Ft1* single mutants, suggesting that these traits are at least in part caused by the p53‐mediated response to the DNA damage elicited by mutation in *Ft1*.

## 2 RESULTS

### 2.1 Generation of *Ft1 kof* mice and characterization of derived MEFs

**Figure 1:**
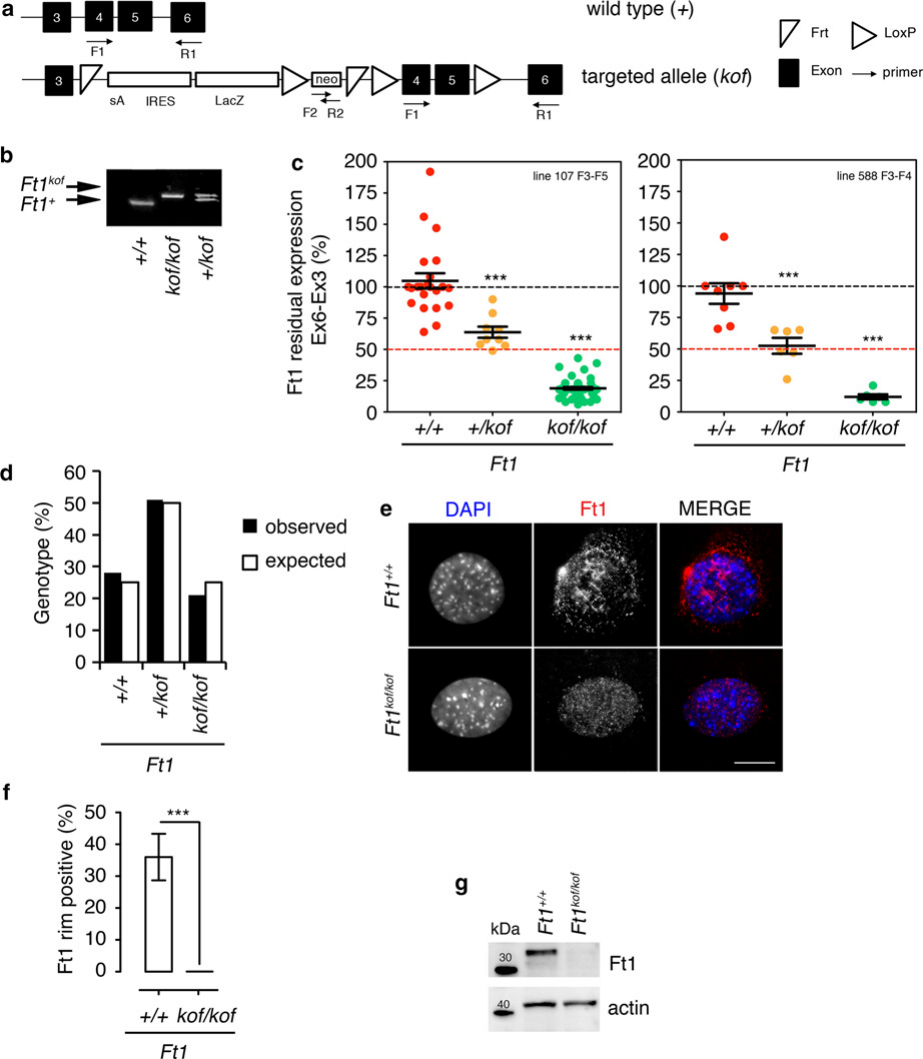
Generation of *Ft1* ^*kof/kof*^ mice. (a) wt allele (+) of *Ft1* and *kof* cassette inserted into the gene to generate animals with reduced *Ft1* expression. F, forward; R, reverse; Frt, target site for FLP recombinase; loxP, target site for Cre recombinase; SA, splicing acceptor site element from *engrailed 2*; IRES, internal ribosomal entry site from the Encephalomyocarditis virus; lacZ, β‐galactosidase gene; neo, neomycin phosphotransferase, selectable marker. (b) PCR of gDNA from *Ft1* ^*+/+*^ *, Ft1* ^*+/kof*^, and *Ft1* ^*kof/kof*^ animals. (c) q‐PCR on tail cDNA of animals from two independent mouse lines. Results present the ratios between *Ft1* exons 6 and 3 normalized to GAPDH. *Ft1* ^*kof/kof*^ mice *n* = 53, *Ft1* ^*+/kof*^ animals *n* = 15, and wt *n* = 30. ***p < .001 in Student's *t* test. (d) Mendelian distribution; no significant difference between observed and expected *Ft1* ^*kof/kof*^ genotypes (*p* = .056 χ^2^ test). (e) MEFs stained with anti‐Ft1 antibody (red in merges). Scale bar 5 μm. (f) Frequency of MEF nuclei showing Ft1 localization at the nuclear rim. Graphs show mean ± SEM;* n* = 50 cells; ***p < .001 in Student's *t* test. (g) Western blotting on MEF extracts

Given that AKTIP is required for DNA replication and cell proliferation (Burla et al., 2015), we reasoned that full knockout (ko) of *Ft1* would cause physiological damage incompatible with mouse survival. Thus, we produced animals with reduced Ft1 levels using the knockout first (kof) strategy, based on the insertion into the target gene (referred as kof allele) of the βgeok cassette (Testa et al., 2004) (Figure 1a), which traps and truncates *Ft1* nascent transcript reducing the expression of the gene (Figure 1a). Transgenic founders and subsequent generations were screened by PCR (Figure 1b), and two independent kof lines (lines 107 and 588) were selected and analyzed for mRNA reduction. q‐PCR on tail biopsies from *Ft1* ^*kof/kof*^ and *Ft1* ^*+/kof*^ animals showed that *Ft1* expression was significantly reduced compared to wild‐type (wt) mice (Figure 1c). In *Ft1* ^*kof/kof*^ mice from lines 107 and 588, *Ft1* expression was reduced to 18% and 12%, respectively; in *Ft1* ^*+/kof*^ animals from the same lines, Ft1 expression was reduced to 64% and 52% (Figure 1c). The analysis of 573 F1 progeny from crosses between Ft1^*+/kof*^ conformed to Mendelian ratios, although we observed a slight nonsignificant trend of embryonic lethality of *Ft1* ^*kof/kof*^ animals (Figure 1d).

We next investigated whether MEFs from *Ft1* ^*kof/kof*^ mice exhibit the same phenotypes as those previously observed in RNAi cells depleted of AKTIP or Ft1 (Burla et al., 2015; Burla, Carcuro, et al., 2016). We first checked the Ft1 subcellular localization by immunostaining MEFs with an anti‐Ft1 antibody. In human cells, AKTIP is enriched at the nuclear rim where it partially co‐localizes with lamins (Burla, Carcuro, et al., 2016). Consistent with these results, *Ft1* ^*+/+*^ MEFs displayed a Ft1 signal at the nuclear periphery (Figure 1e), while the signal was undetectable in the *Ft1* ^*kof/kof*^ MEFs (Figure 1e,f). In line with these results, Western blotting showed a strong reduction of Ft1 in *Ft1* ^*kof/kof*^ MEF extracts (Figure 1g).

**Figure 2:**
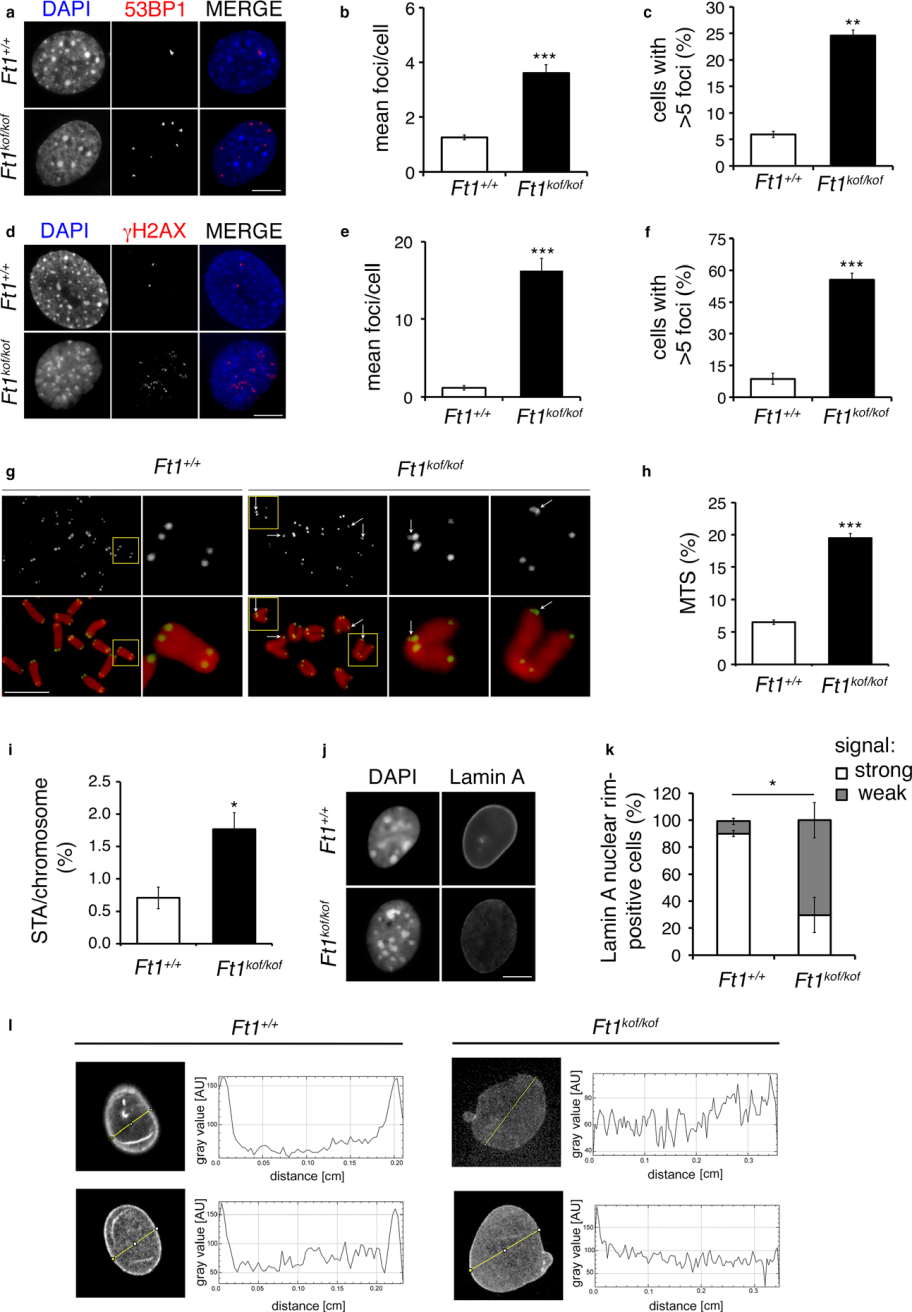
DNA damage, telomere aberrations and lamin A alterations in *Ft1* ^*kof/kof*^ MEFs. (a–c) Staining for anti‐53BP1 (red in merges) in MEFs (a) and quantification (b, c). (d–f) Staining with anti‐γH2AX (red in merges) in MEFs (d) and quantification (e, f). Graphs (b, c, e, f) show mean ± SEM; **p < .01; ***p < .001 in Student's *t* test. Scale bars 5 μm. (g) Partial DAPI‐stained (red) metaphases from MEFs showing telomeric FISH signals (black and white; green in merges) including enlargement of single chromosomes with multiple telomeric signals (MTS). MTS are indicated by arrows. (h, i) MTS (h) and STA (i) frequencies in MEFs. Graphs show mean ± SEM; *p < .05; ***p < .001 in χ^2^ test. (j) Immunostaining for lamin A in MEF nuclei. Scale bar 5 μm. (k) Quantification of lamin A signal at nuclear rim in MEFs. Graphs show mean ± SEM; *p < .05 in Student's *t* test from two independent experiments on two MEF cultures (*n* = 100 cells/culture). (l) Z stack projections and quantification showing the altered distribution of lamin A in *Ft1* ^*kof/kof*^ MEF nuclei. See also Figure S1

We then asked whether MEFs from *Ft1* ^*kof/kof*^ mice activate the DNA damage response (DDR) and exhibit telomere defects. Compared to wt MEFs, *Ft1* ^*kof/kof*^ MEFs displayed substantial increases in 53BP1 and γH2AX foci, indicating that Ft1 is required for the maintenance of genome integrity (Figure 2a–f). In addition, double immunofluorescence staining of γH2AX and TRF1 showed that *Ft1* ^*kof/kof*^ MEFs exhibit a significant increase in γH2AX/TRF1 co‐labeled foci (Telomere Dysfunction induced Foci, TIFs) compared to control MEFs, suggesting that the DDR of mutant cells was at least in part linked to telomere dysfunction (Figure S1c,d). To determine the nature of telomere defects in *Ft1* ^*kof/kof*^ MEFs, we performed in situ hybridization with a TTAGGG probe. The analysis of metaphase spreads showed that *Ft1* ^*kof/kof*^ MEFs exhibit multiple telomeric signals (MTS, also known as fragile telomeres) and sister telomere associations (STA) (Figure 2g–i and Figure S1e). These types of telomere aberrations are considered hallmarks of defective telomere replication (Sfeir et al., 2009). *Ft1* ^*kof/kof*^ MEFs showed a small but not statistically significant increase in telomere fusions compared to matched control MEFs. In addition, *Ft1* ^*kof/kof*^ MEFs displayed a frequency of telomeres with a TTAGGG signal comparable to that of control, confirming (Burla et al., 2015) that an impairment of the Ft1 function does not result in telomere loss (Figure S1a,b).

Finally, we evaluated the status of lamin A in MEFs. Consistent with our previous results on human AKTIP (Burla, Carcuro, et al., 2016), wt cells displayed partial co‐localization of lamin A with Ft1 (Figure S1f). *Ft1* ^*kof/kof*^ MEFs cells showed an altered lamin A distribution with a reduced concentration of lamin A at the nuclear rim (Figure 2j–l).

Altogether, our results indicate that *Ft1* ^*kof*^ mutation cause DDR, telomere defects and abnormal lamin distribution, which are well‐known hallmarks of aging. We thus asked whether *Ft1* ^*kof*^ mutant mice exhibit signs of premature aging. In mouse models of progeroid disorders, premature aging alterations mostly affect body growth, fertility, bones, skin and heart. We therefore focused on these phenotypic traits in our analyses on *Ft1* ^*kof*^ mutant mice.

### 2.2 *Ft1 kof* mice display growth defects, reduced lifespan and sterility

**Figure 3:**
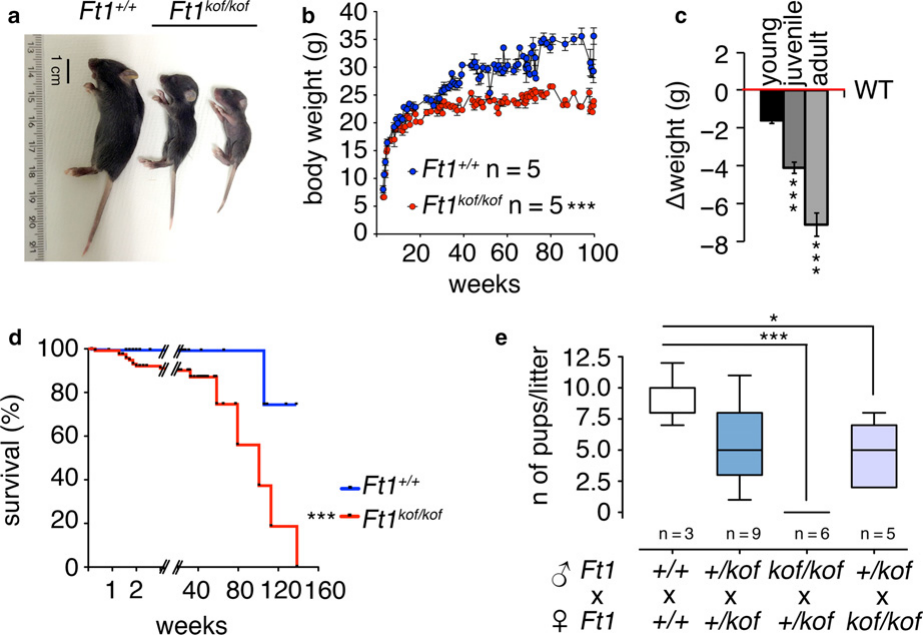
Growth, lifespan, and fertility of *Ft1* ^*kof/kof*^ mice. (a) Pictures of age‐matched mice showing body size differences between *Ft1* ^*kof/kof*^ animals and wt. (b) Body weight analysis during growth of *Ft1* ^*kof/kof*^ and wt. Student's *t* test ***p < .001. (c) Δ(*C*)_t_ in lifespan intervals showing that difference in body weight increases with mouse age; ***p < .001 in Student's *t* test. (d) Kaplan–Meier survival curve of *Ft1* ^*kof/kof*^ and wt mice; ***p < .001—log‐rank—Mantel–Cox test. (e) Pups generated by mice of different genotypes. Whiskers represent the minimum and the maximum values and the boxes the 25th to the 75th percentile; median values are shown as a line within the boxes. *p < .05; ***p < .001 in Student's *t* test. See also Table S2

Macroscopic observation of *Ft1* ^*kof/kof*^ mice revealed that mutant animals (*n* = 170) display a significant reduction in body weight compared to controls (Figure 3a–c); 21% of the animals showed a 30% reduction in body weight compared to controls; henceforth, we will refer to these mice as severely affected *Ft1* ^*kof/kof*^ mice, abbreviated with SA *Ft1* ^*kof/kof*^ or SA mutant mice. By selecting a cohort of animals with a mild (non‐SA) phenotype, we monitored body weight over a 100‐week period and subdivided lifespan in three major intervals: young 3 < weeks < 20; juvenile 21 < weeks < 60; adult 61 < weeks < 100. We observed that the difference in body weight between wt and *Ft1* ^*kof/kof*^ animals significantly increases as mice age (Figure 3b,c). *Ft1* ^*kof/kof*^ mice had a reduced lifespan compared to wt, SA *Ft1* ^*kof/kof*^ animals died at day 12–14, while the remaining population displayed a median survival of 113 weeks (Figure 3d). We also observed reduced fertility in inbreeding; when *Ft1* ^*kof/kof*^ non‐SA males were crossed with *Ft1* ^*+/kof*^ females, we did not observe any pregnancies (Figure 3e). Altogether, these observations show that Ft1 expression is critical for mouse growth, survival and fertility.

### 2.3 *Ft1 kof* mice display skeletal alterations

**Figure 4:**
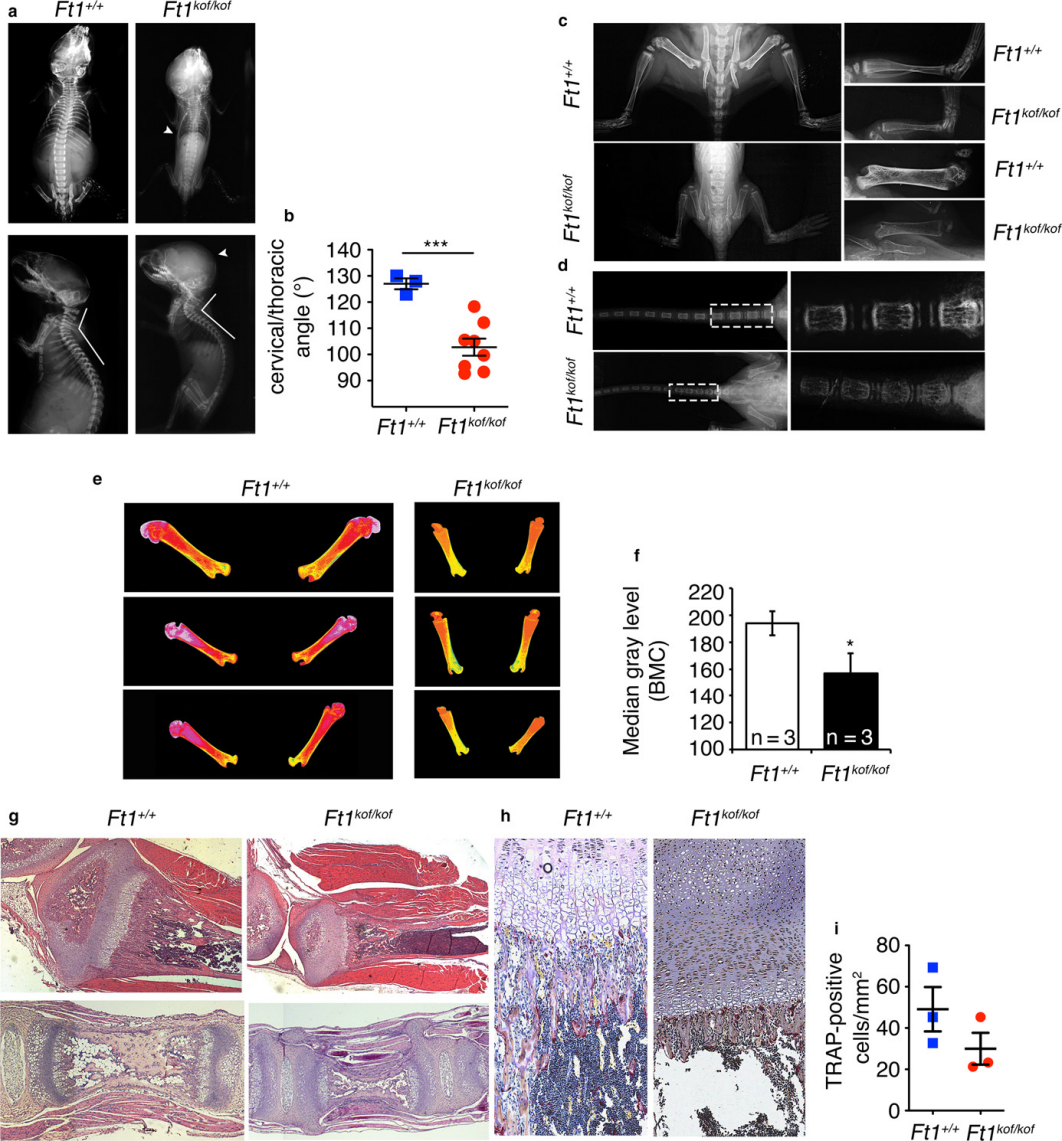
Bone alterations in *Ft1* ^*kof/kof*^ mice. (a) X‐ray on total body of *Ft1* ^*kof/kof*^ and wt mice at day 12. Arrowheads indicate spine defects and craniofacial dysmorphisms in *Ft1* ^*kof/kof*^ animals. (b) Quantification of the angle formed by the cervical and thoracic vertebrae; ***p < .001 in Student's *t* test. (c) X‐ray images of femurs and tibias from *Ft1* ^*kof/kof*^ and wt at day 12. (d) X‐ray of tail and magnification of caudal vertebrae (dotted white box) of *Ft1* ^*kof/kof*^ and wt at day 12. (e, f) Pseudocolored femur images (e) and relative quantification (f) showing that X‐ray absorption is lower in *Ft1* ^*kof/kof*^ as compared to wt. Student's *t* test, *p < .05. (g) H&E‐stained sections of caudal vertebrae (top) and femurs (bottom) of *Ft1* ^*kof/kof*^ and wt at day 12. (h, i) TRAP (h) and relative quantification (i) on femur sections shows no significant differences in TRAP‐positive cells between *Ft1* ^*kof/kof*^ and matched wt (*p* = .67 Student's *t* test). See also Table S2

Bone is altered in progeroid patients and mouse models for progeria syndromes (Bergo et al., 2002; Mounkes, Kozlov, Hernandez, Sullivan & Stewart, 2003; Osorio, Navarro, et al., 2011). Radiographic analyses of whole skeletons were collected at day 12 from eight SA *Ft1* ^*kof/kof*^ mice and three wt animals. X‐ray images showed reduced skeleton size, craniofacial dysmorphism (Figure 4a), and kyphotic spine curvature (Figure 4a,b). Long bones in SA mutant mice were 70% of wt, consistent with the overall body size reduction in *Ft1* ^*kof/kof*^ mice (Figure 4c). This difference is evident in the magnification of femur and tibia (Figure 4c). A reduction in size was also observed in tail vertebrae (Figure 4d). Radiographic analysis further showed that femurs and tibiae from *Ft1* ^*kof/kof*^ mice are less reflective than wt bones. Quantification of pseudocolored images from X‐ray images revealed a statistically significant difference between *Ft1* ^*kof/kof*^ and wt mice, suggesting osteopenic defects in mutant animals (Figure 4e,f).

To further define bone tissue organization, we histologically analyzed sections from caudal vertebrae obtained from SA *Ft1* ^*kof/kof*^ mice. Hematoxilyn and eosin (H&E) staining showed differences in the growth plate of *Ft1* ^*kof/kof*^ animals compared to wt (Figure 4g), while the marrow cavity appeared regularly formed in mutant mice. The analysis of femur sections showed that *Ft1* ^*kof/kof*^ mice exhibit regular columnar and conjugational cartilage, although slightly shorter than controls (Figure 4g). In mutant mice, newly formed bone trabeculae were also shorter, with a poorly defined osteoblastic rim, as compared to wt (Figure 4h). TRAP cytochemistry did not reveal significant differences in osteoclast numbers relative to bone surfaces between *Ft1* ^*kof/kof*^ samples and controls, suggesting that the osteopenic defects cannot be ascribed to increased osteoclastogenesis (Figure 4i).

Altogether, these results show that mutations in *Ft1* cause bone defects that partially phenocopy those observed in progeroid models caused by mutations in lamin coding genes or in genes involved in DNA metabolism (Bergo et al., 2002; Chen et al., 2012; Saeed et al., 2011).

### 2.4 *Ft1 kof* animals display skin and heart alterations

**Figure 5:**
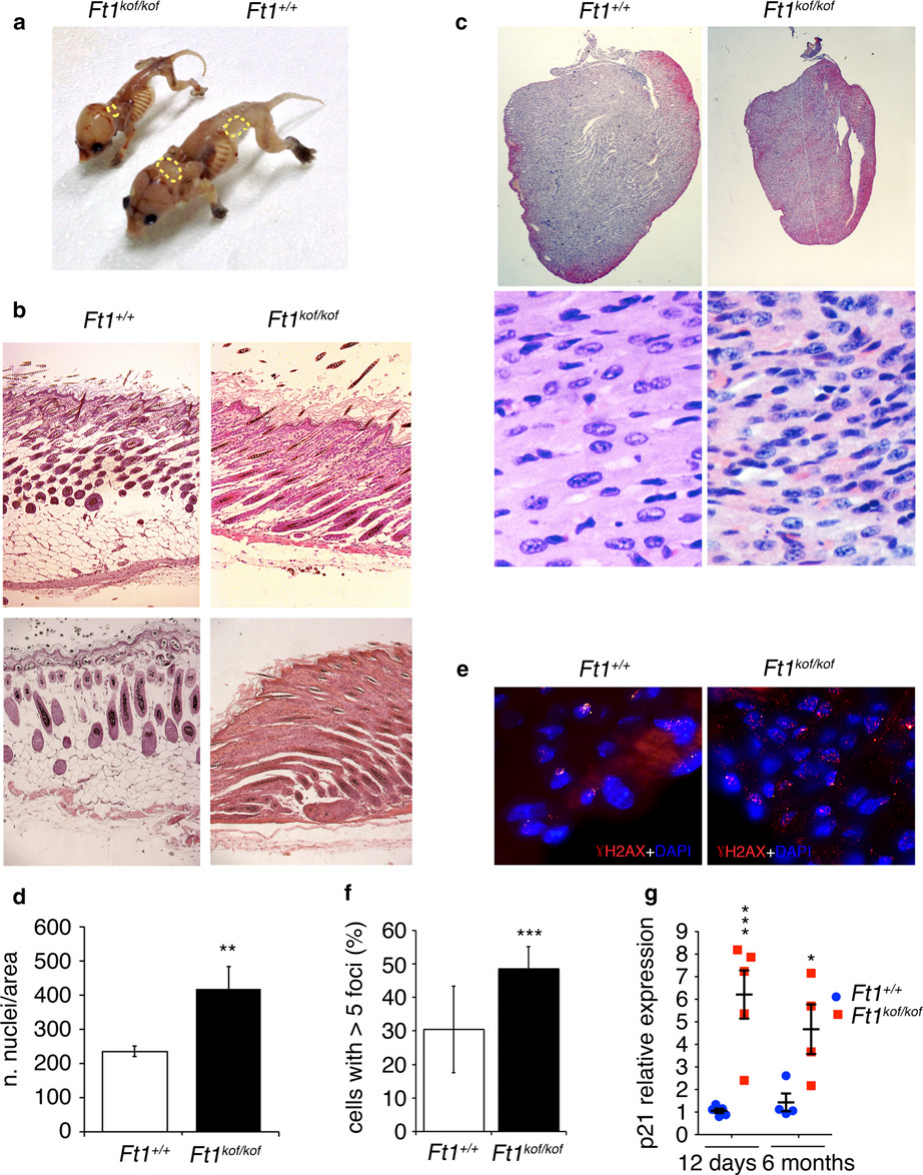
Lipodystrophy and heart defects in *Ft1* ^*kof/kof*^ mice. (a) Pictures of skinned *Ft1* ^*kof/kof*^ and wt at day 12, showing loss of body fat (yellow dot circles) in *Ft1* ^*kof/kof*^ animals. (b) H&E‐stained skin sections of *Ft1* ^*kof/kof*^ and wt at day 12 showing reduction in fat layer. (c) H&E‐stained hearts from wt and *Ft1* ^*kof/kof*^ mice (top) at day 12, and magnification of the heart tissue (bottom). (d) Quantification showing that *Ft1* ^*kof/kof*^ hearts have a higher number of nuclei per area compared to wt; **p < .01 in Student's *t* test. (e) Images of γH2AX immunostaining of heart sections from wt and *Ft1* ^*kof/kof*^ mice at day 12. (f) Percentages of cells showing more than five γH2AX foci in the heart sections shown in e. Error bars indicate SEM; ***p < .001 in Student's *t* test. (g) q‐PCR quantification of the p21 senescence marker expression in wt and matched *Ft1* ^*kof/kof*^ mice at day 12 and at 6 months after birth. *p < .05 and ***p < .001 in Student's *t* test. See also Table S2

Several studies have shown that skin and heart are typically altered in premature aging disorders associated with impaired DNA metabolism, lamin, or telomere defects (Bergo et al., 2002; Cao & Hegele, 2003; Mounkes et al., 2003; Watson et al., 2013). We found that in SA *Ft1* ^*kof/kof*^ mice adipose tissue deposits are strongly reduced compared to age‐matched controls (Figure 5a). The analysis on H&E‐stained skin sections clearly showed the absence of subcutaneous fat layer in SA mutant animals, a defect similar to the skin defects described in mice carrying mutations in the lamin A coding gene (Mounkes et al., 2003) (Figure 5b).

The heart of SA *Ft1* ^*kof/kof*^ mice was smaller than in controls, with a reduction in size proportional to the overall body reduction (Figure 5c). In addition, analysis of H&E‐stained hearts of *Ft1* ^*kof/kof*^ animals and wt mice showed a difference in tissue architecture. In hearts of SA *Ft1* ^*kof/kof*^ animals, there was no apparent fibrotic tissue and the number of nuclei per area was higher than in wt hearts, suggesting an increase in the nuclear/cytoplasmic ratio (Figure 5c,d).

To gain additional insight into the origin of the defects detected in SA *Ft1* ^*kof/kof*^ hearts, we immunostained heart sections for γH2AX to reveal DNA damage foci. In mutant hearts, the frequency of cells with more than 5 foci was significantly higher than in controls, indicating DDR activation (Figure 5e,f). We also investigated whether mutant hearts express the p21 DDR and senescence marker to a higher extent than to control hearts. q‐PCR analysis revealed that *p21* is upregulated in heart extracts from both 12 days SA mutants and 6‐month non‐SA *Ft1* ^*kof/kof*^ mice compared to age‐matched controls (Figure 5g).

Collectively, these results indicate that mutations in *Ft1* affect the skin and heart structural organization, and activate DDR and senescence pathways.

### 2.5 *p53* and *Ft1* genetically interact

p21 is a potent inhibitor of cyclin‐dependent kinase (CDK) that mediates p53‐dependent cell cycle arrest in response to DNA damage; it has been shown that p21 is activated by p53. We thus asked whether p53 contributes to the phenotypic traits observed in *Ft1* ^*kof/kof*^ mice. To test this possibility, we generated *Ft1;p53* double mutant using *p53* ko mice (Jacks et al., 1994). Double‐mutant mice were examined for several phenotypic traits, particularly those affected in *Ft1* ^*kof/kof*^ single mutants.

**Figure 6:**
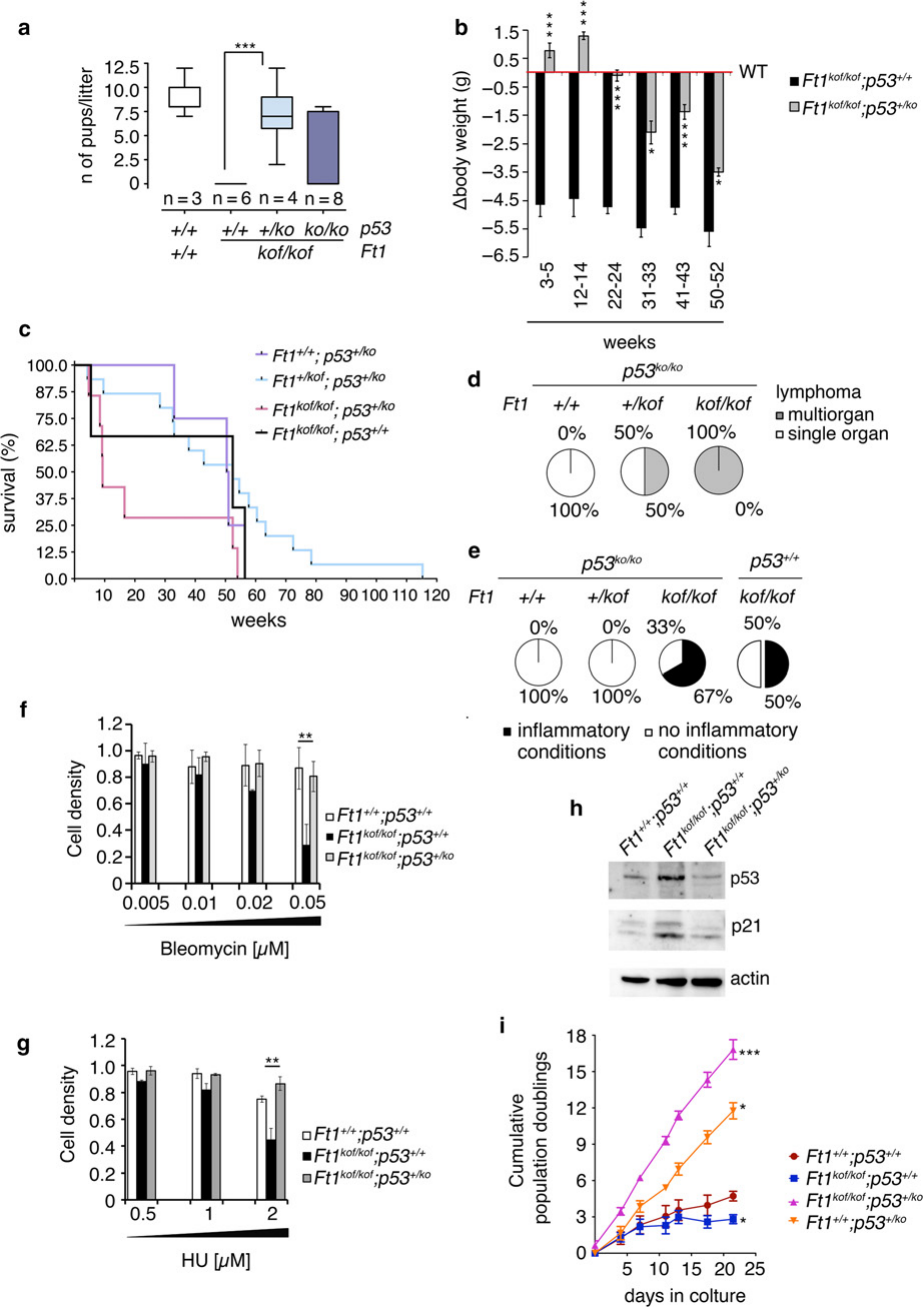
*Ft1* ^*kof/kof*^ mouse phenotype is p53 sensitive. (a) Pups generated by animals bearing mutations in *Ft1* and/or *p53*; ***p < .001 in Student's *t* test. Whiskers represent the minimum and the maximum values observed for each mating and the boxes the 25th to the 75th percentile. (b) Body weight in *Ft1* ^*kof/kof*^ animals in the presence or absence of a null mutation in *p53*; note that loss of a single p53 allele dominantly rescues the *Ft1*‐dependent body weight reduction; *p < .05; ***p < .001 in Student's *t* test. (c) Survival of *Ft1* ^*kof/kof*^ *;p53* ^*+/ko*^ mice is decreased compared to that of *Ft1* ^*+/kof*^ *;p53* ^*+/ko*^ and *Ft1* ^*+/+*^ *;p53* ^*+/ko*^ animals (*p* < .001—log‐rank—Mantel–Cox test). (d, e) Case analysis on wt mice and mice bearing mutations in *p53 (p53* ^*ko/ko*^ *)* and *Ft1,* showing that *Ft1* mutation impacts on lymphomagenesis and inflammatory conditions. See also Figure S3 and Table S1. (f, g) Cell survival response in MEFs from *Ft1* ^*+/+*^ *;p53* ^*+/+*^ *, Ft1* ^*kof/kof*^ *;p53* ^*+/+*^, and *Ft1* ^*kof/kof*^ *;p53* ^*+/ko*^ mice upon increasing doses of bleomycin (f) or hydroxyurea (HU) (g) showing that cells homozygous for mutations in *Ft1* and bearing a null mutation in *p53 (p53* ^*+/ko*^ *)* are less sensitive to DNA damage than *Ft1* mutant cells bearing two wt copies of *p53*. Graphs show mean ± SEM; **p < .01 in Student's *t* test. (h) Western blotting analysis of p21 and p53 expression in *Ft1* ^*+/+*^ *; p53* ^*+/+*^ *, Ft1* ^*kof/kof*^ *; p53* ^*+/+*^, and *Ft1* ^*kof/kof*^ *;p53* ^*+/−*^. (i) Population doubling (pd), showing that *Ft1* ^*kof/kof*^ MEFs have a reduced pd compared to *Ft1* ^*+/+*^ cells; this phenotype is rescued by a *p53* ^*ko*^ mutation. Each dot represents the mean ± SEM of the cumulative pd at the indicated day; *p < .05, ***p < .001 in Student's *t* test. See also Figures S4 and S5

We first analyzed fertility of mutant animals. In contrast with *Ft1* ^*kof/kof*^ male mice that were sterile, *Ft1* ^*kof/kof*^; *p53* ^*+/ko*^ and *Ft1* ^*kof/kof*^ *; p53* ^*ko/ko*^ male mice gave progeny when crossed to wt females, indicating that mutation in one or both *p53* alleles rescues sterility (Figure 6a). We next examined body weight and survival; the body weight deficiency observed in *Ft1* ^*kof/kof*^ mice was rescued in *Ft1* ^*kof/kof*^ *;p53* ^*+/ko*^ mutants at least until the 24th postnatal week. However, after the 31st week, the body weight of *Ft1* ^*kof/kof*^ *;p53* ^*+/ko*^ mutants was reduced compared to controls, although to a lesser extent than in *Ft1* ^*kof/kof*^;*p53* ^*+/+*^ (Figure 6b). Interestingly, *Ft1* ^*kof/kof*^ *;p53* ^*+/ko*^ animals did not exhibit an improvement in their viability as compared to *Ft1* ^*kof/kof*^ *;p53* ^*+/+*^ *;* rather, the double mutation resulted in additive lifespan reduction (Figure 6c).

Analysis of death causes revealed a further interplay between *Ft1* and *p53* (Figure 6d,e and Figure S3). Homozygosity for *Ft1* ^*kof*^ did not result in malignant tumors, and *Ft1* ^*kof*^ mutations were modestly cancer‐protective in a *p53* ko background (Table S1 and Figure S3). However, the simultaneous presence of mutations in *Ft1* and of *p53 loss* induced multiorgan lymphomas (Figure 6d, Figure S3b–e), which were not observed in *p53* mutant animals that exhibit lymphomas in single organs (Figure 6d, Figure S3f,g and Table S1). In addition Mice with reduced levels of Ft1, both in the presence or absence of p53, appeared to be sensitive to other pathologies, including hepatitis, bone marrow aplasia, peritonitis, nephritis, and pneumonia (Figure 6e, and Table S1 and Figure S3h,i). Thus p53 deficiency in *Ft1* ^*kof/kof*^ mutant mice rescues the sterility and the reduced body weight phenotypes, but a concomitant deficiency of *p53* and *Ft1* affects lymphomagenesis.

### 2.6 *Ft1* mutant cells are sensitive to DNA damaging agents

The DDR foci observed in the MEFs and heart of *Ft1* ^*kof/kof*^ mice, and the telomeric aberrations found in *Ft1* ^*kof/kof*^ MEFs suggest that *Ft1* mutant cells might be defective in DNA repair. To address this issue, we determined the sensitivity of *Ft1* ^*kof/kof*^ MEFs to DNA damaging agents. We exposed *Ft1* ^*kof/kof*^ MEFs to nonlethal doses of the radiomimetic compound bleomycin, which creates DNA double‐strand breaks (DSBs). Cell density assessment at 10 days after treatment showed that *Ft1* ^*kof/kof*^ MEFs are significantly more sensitive to the drug compared to passage‐matched wt MEFs (Figure S4a). Increased sensitivity of *Ft1* ^*kof/kof*^ MEFs to DNA damage was also observed after treatment with hydroxyurea, which depletes the cells of dNTPs, generating stalled replication forks that can collapse into DSBs (Figure S4b). Notably, the reduction in cell density observed in *Ft1* ^*kof/kof*^ MEFs after bleomycin or hydroxyurea treatment was rescued by the presence of a single *p53* ^*ko*^ mutant allele in the *Ft1* ^*kof/kof*^ background (Figure 6f,g). In line with these results, the Western blotting analysis showed that nonmutagenized *Ft1* ^*kof/kof*^ MEFs accumulate both p21 and p53 and that this accumulation was significantly reduced in *Ft1* ^*kof/kof*^ *;p53* ^*+/ko*^ MEFs (Figure 6h and Figure S5a,b). Consistent with the finding that p53 and p21 accumulation is associated with cell senescence and reduced proliferation (Ibrahim et al., 2013; Sharpless & Sherr, 2015), we observed a decrease in proliferation of *Ft1* ^*kof/kof*^ MEFs compared to wt MEFs. We also observed an excessive doublings of *Ft1* ^*kof/kof*^ *;p53* ^*+/ko*^ MEFs with respect to *Ft1* ^+/+^ *;p53* ^*+/ko*^ cells (Figure 6i). An increase in the proliferation rate of MEFs bearing mutation in *p53* has been reported previously (Lang et al., 2004; Ma, Choudhury, Hua, Dai & Li, 2013).

Collectively, these results suggest that Ft1 deficiency renders cells more sensitive to DNA damaging agents, resulting in proliferation defects that are (over) rescued by the presence of a single *p53* ^*ko*^ mutant allele.

## 3 DISCUSSION

Human progeroid syndromes and their related animal models have been instrumental to identify factors involved in normal human aging. The cellular defects found in progeroid diseases that also characterize normal human aging include DNA damage and genome instability, telomere attrition, epigenetic alterations of histones, aberrations in the nuclear lamina, and cell senescence (de Boer et al., 2002; Liu et al., 2005; Osorio, Ugalde, et al., 2011; Varela et al., 2005).

Here we analyzed the cellular, developmental, and physiological phenotypes of *Ft1* mutant mice, focusing on traits related to the aging process. Importantly, our analysis of MEFs from *Ft1* ^*kof/kof*^ mice confirmed and extended our previous results obtained on the mouse and human cells depleted of Ft1 or AKTIP (Burla et al., 2015; Burla, Carcuro, et al., 2016). Specifically, we showed that *Ft1* ^*kof/kof*^ mutant MEFs exhibit fragile telomeres and sister telomere associations, TIFs, DNA repair foci, increased sensitivity to bleomycin and hydroxyurea, and reduced cell proliferation. In addition, we confirmed that in *Ft1* mutant MEFs there is an alteration in lamin A, resulting in a strong reduction in the lamin nuclear rim. Thus, *Ft1* mutant MEFs display many traits that have been previously observed in progeroid syndromes and progeroid animal models, as well as in normal human aging.

Consistent with the results on mutant MEFs, our analysis of *Ft1* ^*kof/kof*^ mutant animals detected progeroid phenotypes. *Ft1* ^*kof/kof*^ mice displayed multiple traits that have been previously observed in several progeroid models (Table S2). We found that *Ft1* mutant mice have reduced body weight, fertility defects, and reduced lifespan, as previously observed in models of laminopathies (Bergo et al., 2002; Osorio, Navarro, et al., 2011) and telomeropathies (Martínez et al., 2009). In addition, the growth defects of *Ft1* ^*kof/kof*^ mice were exacerbated with aging, suggesting that the effects of *Ft1* mutations intercept the normal aging‐induced degeneration pathways. *Ft1* ^*kof/kof*^ mice also displayed skin and bone defects, which were previously observed in lamin mutant mice (Bergo et al., 2002; Mounkes et al., 2003; Osorio, Navarro, et al., 2011), in *Tert* ko animals (Rudolph et al., 1999), and in mice with reduced Trf1 expression (Martínez et al., 2009). Skeletal alterations and lipodystrophy have been imputed to failures in the proliferation of mesenchymal stem cell progenitors, which are sensitive to lamin mutations and senescence (Scaffidi & Misteli, 2008). Mutant hearts were smaller than those of wt animals and showed a higher nuclear density compared to wt, with an increase in the nuclear/cytoplasmic ratio. In addition, we found that mutant hearts display DNA damage and activation of the DDR, and up‐regulation of p21 expression. The relationships between increased DNA damage and a change in nuclear density in the mutant hearts are unclear. A possible explanation is that DNA damage and the related inflammation process induce local cell reprogramming. This explanation is consistent with the observation that cellular reprogramming in vivo occurs following tissue injury (Yanger et al., 2013).

The fact that the organismal phenotypes observed in *Ft1* mutant animals have also been found in models specifically defective in lamin structure and/or expression, or bearing mutations in genes required for telomere maintenance or DNA repair, poses an interesting question. Which of the cellular phenotypes observed in *Ft1* mutant MEFs (defective lamin behavior, telomere dysfunction, DNA damage) is responsible for the organismal progeroid phenotypes? Answering this question is difficult because the traits that characterize *Ft1* mutants at the cellular level are deeply interconnected. For example, alterations in lamin function affect DNA replication and repair, epigenetic modification of chromatin and transcription (Gonzalo & Kreienkamp, 2015). Moreover, multiple interactions link telomeres to the lamin network, including the association of telomeres with the nuclear envelope (Burla, La Torre & Saggio, 2016). Finally, telomeres recruit and interact with many DNA repair factors, which play crucial functions in telomere maintenance (Doksani & de Lange, 2014). Thus, current information does not allow identification of the specific cellular phenotype that leads to progeroid traits observed in *Ft1* mutant mice. The most likely hypothesis is that all cellular defects observed in *Ft1* mutant MEFs contribute to the organismal phenotype of mutant animals. It is indeed quite possible that these defects lead to senescence in most if not all tissues, causing developmental defects and infertility.

### 3.1 Relationships between *Ft1*,* p53,* and cancer

We have shown that p53 deficiency in *Ft1* mutant MEFs induces cell over proliferation and rescues the sensitivity to both bleomycin and hydroxyurea. Consistent with these findings, in *Ft1* ^*kof/kof*^ mutant mice, mutations in *p53* rescue the body weight and sterility phenotypes but do not improve survival. Impairment of the p53 function also ameliorates the progeroid phenotypes in BRCA1‐deficient mice (Cao, Li, Kim, Brodie & Deng, 2003) and in HGPS mouse models (Varela et al., 2005). However, p53 deficiency worsens the progeroid phenotype in telomere dysfunctional mice (Begus‐Nahrmann et al., 2009). An explanation for this discrepancy is that p53 deficiency allows beneficial propagation of damaged cells rescuing certain progeroid traits. However, when cellular damage is extensive and the regenerative capacity of tissues is severely limited, p53 deficiency would become deleterious and accelerate aging (Lopez‐Otin, Blasco, Partridge, Serrano & Kroemer, 2013). Our results are consistent with this model; they indicate that mutations in *Ft1* result in a relatively mild genomic damage that triggers DDR‐related checkpoints, which are abolished by mutations in *p53* allowing resumption of cell proliferation.

The relationships between mutations in progeria‐related genes and cancer are also complex. Progeroid models have been used to study the interplay between aging and cancer, given that age is a major risk factor for cancer developing. It has been shown that some forms of progeria can exert a protective role against tumor development (de la Rosa et al., 2013). On the other hand, mutations in the WRN helicase causing a segmental progeroid syndrome have been associated with an elevated cancer risk (Blander et al., 2000). We found that *Ft1^kof^* mutation does not induce cancer and that *p53 Ft1* double‐mutant mice do not exhibit an increase in the overall frequency of malignancies. However, *p53* ko combined with *Ft1* deficiency induced an increase in the diffusion of lymphomas as compared to the restricted localization of this type of tumor in *p53* ko mice. It has been reported that T‐cell lymphomas in *p53* ko mice are oligoclonal and generated by a characterized sequence of mutational events (Dudgeon et al., 2014). We therefore hypothesize that in *p53 Ft1* double mutants, this sequence is altered causing multiclonality and/or histotype change of lymphomas.

In conclusion, we have shown that mutations in Ft1 affect lamin, telomeres, DNA repair, and cell senescence. At the organismal level, these mutations result in a number of phenotypes that have been previously observed in several progeria mouse models. Thus, we believe that Ft1 is a new player in both the normal and accelerated aging processes, and that *Ft1* mutant mice will be instrumental to analyze the interactions between *Ft1* and other mouse progeria genes.

## 4 EXPERIMENTAL PROCEDURES

### 4.1 Mice

ES (HEPD0589_6_H06) from C57Bl/6 animals carrying the knockout first mutations in the *Ft1* gene (referred as *Ft1 kof*) were generated by the International Knock‐out mouse consortium. Injections into C57Bl/6 blastocyst were performed in EMBL (Monterotondo, Italy). Chimeras were crossed with C57Bl/6 and heterozygous animals backcrossed with C57Bl/6 and/or intercrossed. *Ft1* ^*+/kof*^ were crossed with *p53* ^*+/ko*^ (Jacks et al., 1994) animals to obtain double mutants. Offspring were weaned at 3 weeks, and tail biopsies were genotyped and transgene expression analyzed. When needed, mice were anesthetized by intramuscular Zoletil 20 (Virbac S.A., France), or euthanized by asphyxiation with carbon dioxide or cervical dislocation.

### 4.2 Cells

MEFs were isolated and cultured as described in Rinaldo et al. (2012). Population doubling (pd) was calculated with the formula Log(*n*_*t*_/*n*_0_) × 3.33. For Bleomycin and Hydroxyurea sensitivity assay, cells plated 24 hr in advance were treated with Bleomycin (Sanofi Aventis) or Hydroxyurea (Sigma) for 7 hr and replaced with medium w/o drugs. Cell density was calculated 10 days after treatment by staining with crystal violet (5% in methanol, Sigma) for 10 min and analyzed by imagej.

### 4.3 Genotyping

Tail biopsies were digested overnight at 56°C with a proteinase K/SDS solution; genomic DNA (gDNA) was extracted using the NucleoSpin^®^ Tissue columns kit (Macherey‐Nagel, Duren, Germany) following manufacturer's instructions. Mice were PCR genotyped using the following primers:

*Ft1* E4 F: 5′–GTGAAGCAGAAGCTGCCAGGAGT–3′;
*Ft1* E6 R: 5′–AGCTCACCCGAGGTGGGATCAA–3′;
*p53*‐X6 F: 5′–AGCGTGGTGGTACCTTATGAGC–3′;
*p53*‐Neo19 F: 5′–GCTATCAGGACATAGCGTTGGC–3′;
*p53*‐X7 R: 5′–GGATGGTGGTATACTCAGAGCC–3′;

### 4.4 q‐PCR

RNA was extracted using the TRIzol reagent (Invitrogen) according to manufacturer, after DNaseI treatment (Invitrogen) was reverse transcribed into cDNA with oligo d(T) primer and OMNISCRIPT RT KIT (Qiagen). q‐PCRs were performed as described (Burla et al., 2015) using following primers:

*Ft1* E3 F: AACCAGTCCTCCACGAAGTGCA;
*Ft1* E3 R: TAGGGCTTCGCTATGGGTAGAGCA;
*Ft1* E6 F: CCGTCTTTCACCCACTAGTTGAT;
*Ft1* E6 R: TTGCGAACGCTCTTTTCACA;
*mGAPDH* F: GTGGCAAAGTGGAGATTGTTGCC;
*mGAPDH* R: TGTGCCGTTGAATTTGCCGT;
*p21* F: 5′–TGTCTGAGCGGCCTGAAGAT–3′;
*p21* R: 5′–CTGCGCTTGGAGTGATAGAA–3′

### 4.5 Western blotting

Western blotting was carried out as described in Burla et al. (2015). Filters were incubated with rabbit monoclonal anti‐FT1 (Sigma HPA 046300), rabbit anti‐actin HRP‐conjugated (C‐11; Santa Cruz—sc1615), rabbit anti‐p21 (C‐19; Santa Cruz—sc397), and rabbit anti‐p53 antibodies produced by S. Soddu as described in Cecchinelli et al. (2006). Filters were incubated with appropriate HRP‐conjugated secondary antibodies (Santa Cruz).

### 4.6 Immunostaining, FISH, and cytology

For immunostaining, cells were fixed with 3.7% formaldehyde for 10 min at 4°C and permeabilized with 0.25% Triton X‐100 in PBS for 5 min. Where indicated, cells were prepermeabilized according to (Burla et al., 2015). Cells were then incubated with the following antibodies in the presence of 3% BSA: anti‐53BP1 (Novus Biologicals NB100‐304), anti‐γH2AX (05‐636 clone JBW301 Upstate Biotechnology), anti‐Trf1 (Abcam 1423), anti‐Ft1 (Sigma, HPA 046300), and anti‐ Lamin A (H102, Santa Cruz Biotechnology sc 20680). Primary antibodies were detected with the pertinent secondary antibodies: anti‐rabbit‐ALEXA 555 (Invitrogen A21430) or anti‐goat‐FITC (Jackson Immunoresearch 705‐095‐003). FISH was carried out according to Burla et al. (2015). Cytological preparations were examined with a Carl Zeiss (Thornwood, NY) Axioplan fluorescence microscope equipped with an HBO100W mercury lamp and a cooled charged‐coupled device (CCD camera; Photometrics CoolSnap HQ). Optical sections were captured at 0.3 μm Z steps using a Prior Proscan stepping motor with an EM‐CCD camera (Cascade II, Photometrics) connected to a spinning‐disk confocal head (CarvII, Beckton Dickinson) mounted on an inverted microscope (Eclipse TE2000S, Nikon). Each image is a maximum‐intensity projection of all sections. Images were recorded using metamorph software package (Universal Imaging) and processed using imagej (http://imagej.nih.gov) and Adobe Photoshop.

### 4.7 Histology, immunohistochemistry, and TRAP

Skin, bone, and heart were fixed in 4% formaldehyde. Tissues were cleared with ascendant alcohol concentration, embedded in paraffin, and sectioned at 3.5 μm. Sections were hydrated with descendant alcohol concentration, stained with Hematoxylin (Carlo Erba) and Eosin (Sigma), cleared, and mounted with DPX Mountant for Histology (Sigma). For γH2AX analysis on paraffin, embedded heart sections were treated as previously described (Martinez, Ferrara‐Romeo, Flores & Blasco, 2014). Tissues were incubated overnight with an anti‐γH2AX (Abcam 2893) diluted in BSA 3%, Triton X‐100 0.1%, and the day after incubated for 1 hr at room temperature with the pertinent secondary antibody (anti‐rabbit‐ALEXA 555, Invitrogen A21430). Slides were counterstained with Mayer hematoxilin (Carlo Erba) and mounted with DPX mounting solution for microscopic evaluation (Sigma). Pictures were taken with ZEISS‐Axio Phot (Zeiss) microscope connected to Progress‐C5 JENO‐PTIK camera with the software progress mac (Capture PRO). TRAP staining was performed according to manufacturer's instructions (Sigma 387A).

### 4.8 X‐ray and bone density analysis

Total body X‐ray images were taken using Faxitron MX‐20 (Faxitron X‐ray Corp.) at 24 kV for 6 s; images captured with Medical Imaging Film HM Plus (Ferrania). Cervical‐thoracic vertebrae angle quantification was measured with Photoshop CS6 plugin. Femurs were imaged using a Faxitron MX20 operating at 24 kV for 4 s. Image density was determined as previously described (Bassett, van der Spek, Gogakos & Williams, 2012).

### 4.9 Statistics

Kaplan–Meier curves were analyzed using the log‐rank (Mantel‐Cox) test. Inheritance of *kof* allele was analyzed using the Mendelian ratio for heterozygous mating, and χ^2^ test was applied. The Kolmogorov–Smirnov test was used to compare gray‐level cumulative frequency distributions in X‐ray image quantification. Independent data sets were compared with the Student's *t* test (unpaired, two‐tailed).

## CONFLICT OF INTEREST

We have no conflict of interest.

## AUTHOR CONTRIBUTIONS

MLT, CM, RB, GZ, SDG, MC, IV, EB, AB, IM, and GRV performed the experiments. MR, AC, FV, SS, MG, and GP contributed to the design of the experiments and to the writing of the manuscript. IS designed the experiments and wrote the manuscript.

## References

[acel12730-bib-0001] Bachrati, C. Z. , & Hickson, I. D. (2003). RecQ helicases: Suppressors of tumorigenesis and premature aging. Biochemical Journal, 374, 577–606. 10.1042/bj20030491 12803543 PMC1223634

[acel12730-bib-0002] Barrowman, J. , Wiley, P. A. , Hudon‐Miller, S. E. , Hrycyna, C. A. , & Michaelis, S. (2012). Human ZMPSTE24 disease mutations: Residual proteolytic activity correlates with disease severity. Human Molecular Genetics, 21, 4084–4093. 10.1093/hmg/dds233 22718200 PMC3428156

[acel12730-bib-0003] Bassett, J. H. , van der Spek, A. , Gogakos, A. , & Williams, G. R. (2012). Quantitative X‐ray imaging of rodent bone by Faxitron. Methods in Molecular Biology, 816, 499–506. 10.1007/978-1-61779-415-5 22130946

[acel12730-bib-0004] Begus‐Nahrmann, Y. , Lechel, A. , Obenauf, A. C. , Nalapareddy, K. , Peit, E. , Hoffmann, E. , … Rudolph, K. L. (2009). p53 deletion impairs clearance of chromosomal‐instable stem cells in aging telomere‐dysfunctional mice. Nature Genetics, 41, 1138–1143. 10.1038/ng.426 19718028

[acel12730-bib-0005] Bergo, M. O. , Gavino, B. , Ross, J. , Schmidt, W. K. , Hong, C. , Kendall, L. V. , … Young, S. G. (2002). Zmpste24 deficiency in mice causes spontaneous bone fractures, muscle weakness, and a prelamin A processing defect. Proceedings of the National Academy of Sciences USA, 99, 13049–13054. 10.1073/pnas.192460799 PMC130584 12235369

[acel12730-bib-0006] Blander, G. , Zalle, N. , Leal, J. F. , Bar‐Or, R. L. , Yu, C. E. , & Oren, M. (2000). The Werner syndrome protein contributes to induction of p53 by DNA damage. FASEB Journal, 14, 2138–2140. 11023999 10.1096/fj.00-0171fje

[acel12730-bib-0007] Burla, R. , Carcuro, M. , La Torre, M. , Fratini, F. , Crescenzi, M. , D'Apice, M. , … Saggio, I. (2016). The telomeric protein AKTIP interacts with A‐ and B‐type lamins and is involved in regulation of cellular senescence. Open Biology, 6, 160103 10.1098/rsob.160103 27512140 PMC5008010

[acel12730-bib-0008] Burla, R. , Carcuro, M. , Raffa, G. D. , Galati, A. , Raimondo, D. , Rizzo, A. , … Saggio, I. (2015). AKTIP/Ft1, a new shelterin‐interacting factor required for telomere maintenance. PLoS Genetics, 11, e1005167 10.1371/journal.pgen.1005167 26110528 PMC4481533

[acel12730-bib-0009] Burla, R. , La Torre, M. , & Saggio, I. (2016). Mammalian telomeres and their partnership with lamins. Nucleus, 7, 187–202. 10.1080/19491034.2016.1179409 27116558 PMC4916877

[acel12730-bib-0010] Cao, H. , & Hegele, R. A. (2003). LMNA is mutated in Hutchinson‐Gilford progeria (MIM 176670) but not in Wiedemann‐Rautenstrauch progeroid syndrome (MIM 264090). Journal of Human Genetics, 48, 271–274. 10.1007/s10038-003-0025-3 12768443

[acel12730-bib-0011] Cao, L. , Li, W. , Kim, S. , Brodie, S. G. , & Deng, C. X. (2003). Senescence, aging, and malignant transformation mediated by p53 in mice lacking the Brca1 full‐length isoform. Genes & Development, 17, 201–213. 10.1101/gad.1050003 12533509 PMC195980

[acel12730-bib-0012] Cecchinelli, B. , Porrello, A. , Lazzari, C. , Gradi, A. , Bossi, G. , D'Angelo, M. , … Soddu, S. (2006). Ser58 of mouse p53 is the homologue of human Ser46 and is phosphorylated by HIPK2 in apoptosis. Cell Death and Differentiation, 13, 1994–1997. 10.1038/sj.cdd.4401933 16729035

[acel12730-bib-0013] Cenci, G. , Ciapponi, L. , Marzullo, M. , Raffa, G. D. , Morciano, P. , Raimondo, D. , … Gatti, M. (2015). The analysis of pendolino (peo) mutants reveals differences in the fusigenic potential among *Drosophila* telomeres. PLoS Genetics, 11, e1005260 10.1371/journal.pgen.1005260 26110638 PMC4481407

[acel12730-bib-0015] Chen, C. Y. , Chi, Y. H. , Mutalif, R. A. , Starost, M. F. , Myers, T. G. , Anderson, S. A. , … Jeang, K. T. (2012). Accumulation of the inner nuclear envelope protein Sun1 is pathogenic in progeric and dystrophic laminopathies. Cell, 149, 565–577. 10.1016/j.cell.2012.01.059 22541428 PMC3340584

[acel12730-bib-0017] de la Rosa, J. , Freije, J. M. , Cabanillas, R. , Osorio, F. G. , Fraga, M. F. , Fernandez‐Garcia, M. S. , … Lopez‐Otin, C. (2013). Prelamin A causes progeria through cell‐extrinsic mechanisms and prevents cancer invasion. Nature Communications, 4, 2268. 10.1038/ncomms3268 PMC3758871 23917225

[acel12730-bib-0018] De Sandre‐Giovannoli, A. , Bernard, R. , Cau, P. , Navarro, C. , Amiel, J. , Boccaccio, I. , … Levy, N. (2003). Lamin a truncation in Hutchinson‐Gilford progeria. Science, 300, 2055 10.1126/science.1084125 12702809

[acel12730-bib-0019] Dokal, I. (2011). Dyskeratosis congenita. Hematology/the Education Program of the American Society of Hematology, 2011, 480–486. 10.1182/asheducation-2011.1.480 22160078

[acel12730-bib-0020] Doksani, Y. , & de Lange, T. (2014). The role of double‐strand break repair pathways at functional and dysfunctional telomeres. Cold Spring Harbor Perspectives in Biology, 6, a016576 10.1101/cshperspect.a016576 25228584 PMC4292156

[acel12730-bib-0021] Dudgeon, C. , Chan, C. , Kang, W. , Sun, Y. , Emerson, R. , Robins, H. , & Levine, A. J. (2014). The evolution of thymic lymphomas in p53 knockout mice. Genes & Development, 28, 2613–2620. 10.1101/gad.252148.114 25452272 PMC4248292

[acel12730-bib-0022] Gonzalo, S. , & Kreienkamp, R. (2015). DNA repair defects and genome instability in Hutchinson‐Gilford Progeria Syndrome. Current Opinion in Cell Biology, 34, 75–83. 10.1016/j.ceb.2015.05.007 26079711 PMC4522337

[acel12730-bib-0023] Hennekam, R. C. (2006). Hutchinson‐Gilford progeria syndrome: Review of the phenotype. American Journal of Medical Genetics. Part A, 140, 2603–2624. 10.1002/(ISSN)1552-4833 16838330

[acel12730-bib-0024] Ibrahim, M. X. , Sayin, V. I. , Akula, M. K. , Liu, M. , Fong, L. G. , Young, S. G. , & Bergo, M. O. (2013). Targeting isoprenylcysteine methylation ameliorates disease in a mouse model of progeria. Science, 340, 1330–1333. 10.1126/science.1238880 23686339 PMC4295631

[acel12730-bib-0025] Jacks, T. , Remington, L. , Williams, B. O. , Schmitt, E. M. , Halachmi, S. , Bronson, R. T. , & Weinberg, R. A. (1994). Tumor spectrum analysis in p53‐mutant mice. Current Biology, 4, 1–7. 10.1016/S0960-9822(00)00002-6 7922305

[acel12730-bib-0026] Lang, G. A. , Iwakuma, T. , Suh, Y. A. , Liu, G. , Rao, V. A. , Parant, J. M. , … Lozano, G. (2004). Gain of function of a p53 hot spot mutation in a mouse model of Li‐Fraumeni syndrome. Cell, 119, 861–872. 10.1016/j.cell.2004.11.006 15607981

[acel12730-bib-0028] Liu, B. , Wang, J. , Chan, K. , Tjia, W. , Deng, W. , Guan, X. , … Zhou, Z. (2005). Genomic instability in laminopathy‐based premature aging. Nature Medicine, 11, 780–785. 10.1038/nm1266 15980864

[acel12730-bib-0029] Lopez‐Otin, C. , Blasco, M. A. , Partridge, L. , Serrano, M. , & Kroemer, G. (2013). The hallmarks of aging. Cell, 153, 1194–1217. 10.1016/j.cell.2013.05.039 23746838 PMC3836174

[acel12730-bib-0030] Ma, X. , Choudhury, S. N. , Hua, X. , Dai, Z. , & Li, Y. (2013). Interaction of the oncogenic miR‐21 microRNA and the p53 tumor suppressor pathway. Carcinogenesis, 34, 1216–1223. 10.1093/carcin/bgt044 23385064 PMC3670255

[acel12730-bib-0031] Martinez, P. , Ferrara‐Romeo, I. , Flores, J. M. , & Blasco, M. A. (2014). Essential role for the TRF2 telomere protein in adult skin homeostasis. Aging Cell, 13, 656–668. 10.1111/acel.12221 24725274 PMC4326939

[acel12730-bib-0032] Martínez, P. , Thanasoula, M. , Muñoz, P. , Liao, C. , Tejera, A. , McNees, C. , … Blasco, M. A. (2009). Increased telomere fragility and fusions resulting from TRF1 deficiency lead to degenerative pathologies and increased cancer in mice. Genes & Development, 23, 2060–2075. 10.1101/gad.543509 19679647 PMC2751970

[acel12730-bib-0033] Mounkes, L. C. , Kozlov, S. , Hernandez, L. , Sullivan, T. , & Stewart, C. L. (2003). A progeroid syndrome in mice is caused by defects in A‐type lamins. Nature, 423, 298–301. 10.1038/nature01631 12748643

[acel12730-bib-0035] Osorio F. G. , Navarro C. L. , Cadinanos J. , Lopez‐Mejia I. C. , Quiros P. M. , Bartoli C. , … Lopez‐Otin C. (2011) Splicing‐directed therapy in a new mouse model of human accelerated aging. Science Translational Medicine 3, 106ra107. 10.1126/scitranslmed.3002847 22030750

[acel12730-bib-0036] Osorio, F. G. , Ugalde, A. P. , Marino, G. , Puente, X. S. , Freije, J. M. , & Lopez‐Otin, C. (2011). Cell autonomous and systemic factors in progeria development. Biochemical Society Transactions, 39, 1710–1714. 10.1042/BST20110677 22103512

[acel12730-bib-0038] Rinaldo, C. , Moncada, A. , Gradi, A. , Ciuffini, L. , D'Eliseo, D. , Siepi, F. , … Soddu, S. (2012). HIPK2 controls cytokinesis and prevents tetraploidization by phosphorylating histone H2B at the midbody. Molecular Cell, 47, 87–98. 10.1016/j.molcel.2012.04.029 22658722

[acel12730-bib-0039] Rudolph, K. L. , Chang, S. , Lee, H. W. , Blasco, M. , Gottlieb, G. J. , Greider, C. , & DePinho, R. A. (1999). Longevity, stress response, and cancer in aging telomerase‐deficient mice. Cell, 96, 701–712. 10.1016/S0092-8674(00)80580-2 10089885

[acel12730-bib-0040] Saeed, H. , Abdallah, B. M. , Ditzel, N. , Catala‐Lehnen, P. , Qiu, W. , Amling, M. , & Kassem, M. (2011). Telomerase‐deficient mice exhibit bone loss owing to defects in osteoblasts and increased osteoclastogenesis by inflammatory microenvironment. Journal of Bone and Mineral Research, 26, 1494–1505. 10.1002/jbmr.349 21308778

[acel12730-bib-0041] Scaffidi, P. , & Misteli, T. (2008). Lamin A‐dependent misregulation of adult stem cells associated with accelerated ageing. Nature Cell Biology, 10, 452–459. 10.1038/ncb1708 18311132 PMC2396576

[acel12730-bib-0042] Sfeir, A. , Kosiyatrakul, S. T. , Hockemeyer, D. , MacRae, S. L. , Karlseder, J. , Schildkraut, C. L. , & de Lange, T. (2009). Mammalian telomeres resemble fragile sites and require TRF1 for efficient replication. Cell, 138, 90–103. 10.1016/j.cell.2009.06.021 19596237 PMC2723738

[acel12730-bib-0043] Sharpless, N. E. , & Sherr, C. J. (2015). Forging a signature of in vivo senescence. Nature Reviews Cancer, 15, 397–408. 10.1038/nrc3960 26105537

[acel12730-bib-0045] Testa, G. , Schaft, J. , van der Hoeven, F. , Glaser, S. , Anastassiadis, K. , Zhang, Y. , … Stewart, A. F. (2004). A reliable lacZ expression reporter cassette for multipurpose, knockout‐first alleles. Genesis, 38, 151–158. 10.1002/(ISSN)1526-968X 15048813

[acel12730-bib-0046] Varela, I. , Cadinanos, J. , Pendas, A. M. , Gutierrez‐Fernandez, A. , Folgueras, A. R. , Sanchez, L. M. , … Lopez‐Otin, C. (2005). Accelerated ageing in mice deficient in Zmpste24 protease is linked to p53 signalling activation. Nature, 437, 564–568. 10.1038/nature04019 16079796

[acel12730-bib-0047] Watson, L. A. , Solomon, L. A. , Li, J. R. , Jiang, Y. , Edwards, M. , Shin‐ya, K. , … Berube, N. G. (2013). Atrx deficiency induces telomere dysfunction, endocrine defects, and reduced life span. Journal of Clinical Investigation, 123, 2049–2063. 10.1172/JCI65634 23563309 PMC3635723

[acel12730-bib-0049] Yanger, K. , Zong, Y. , Maggs, L. R. , Shapira, S. N. , Maddipati, R. , Aiello, N. M. , … Stanger, B. Z. (2013). Robust cellular reprogramming occurs spontaneously during liver regeneration. Genes & Development, 27, 719–724. 10.1101/gad.207803.112 23520387 PMC3639413

