# Supplementary material for: Mice with reduced expression of the telomere‐associated protein Ft1 develop p53‐sensitive progeroid traits
Source: Aging Cell. 2018 Apr 10;17(4):e12730. doi: 10.1111/acel.12730 (PMC6052474; doi:10.1111/acel.12730)
Supplement: Supplementary file 1 [file ACEL-17-na-s001.docx]

**Supporting Information**

**Supplementary figures**

**Figure S1**


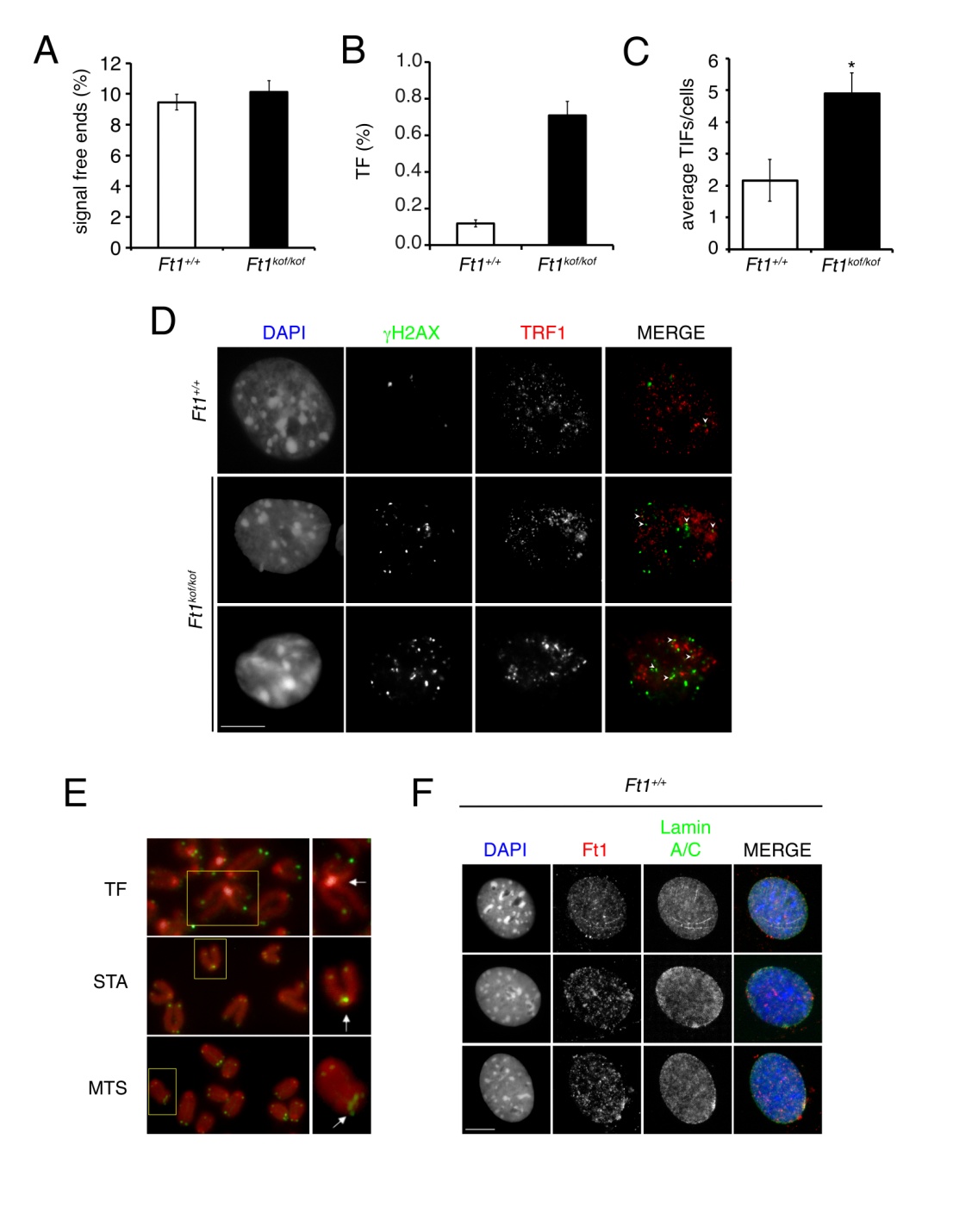


**Figure S2**

**
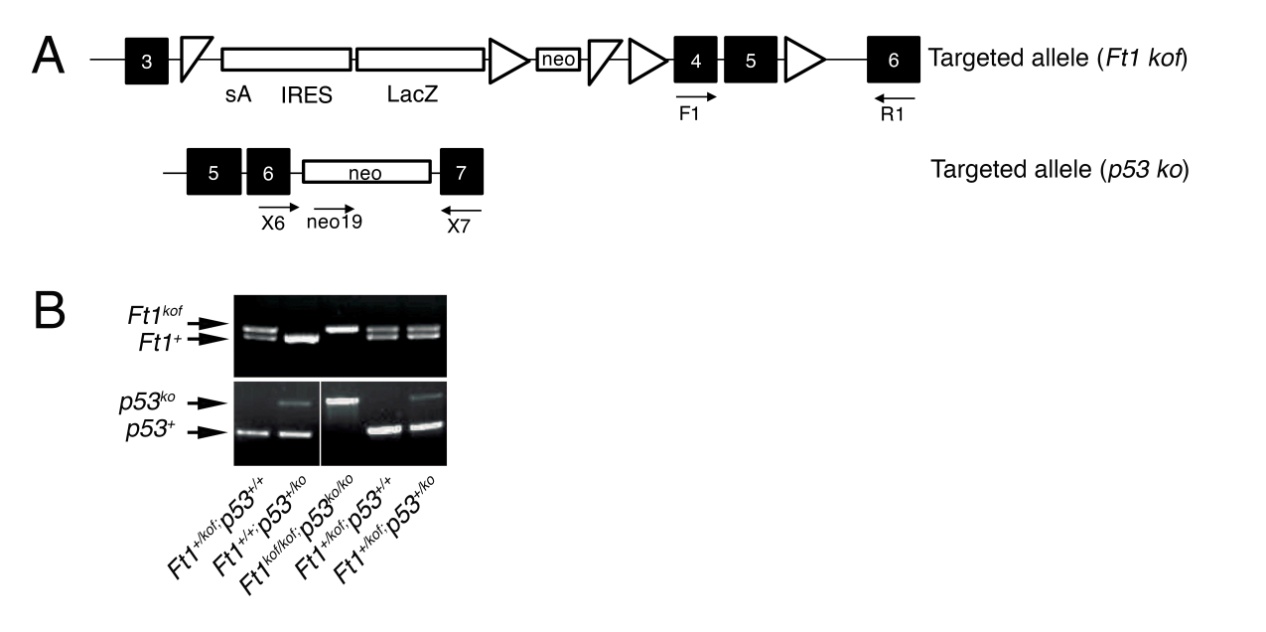
**

**Figure S3**

**
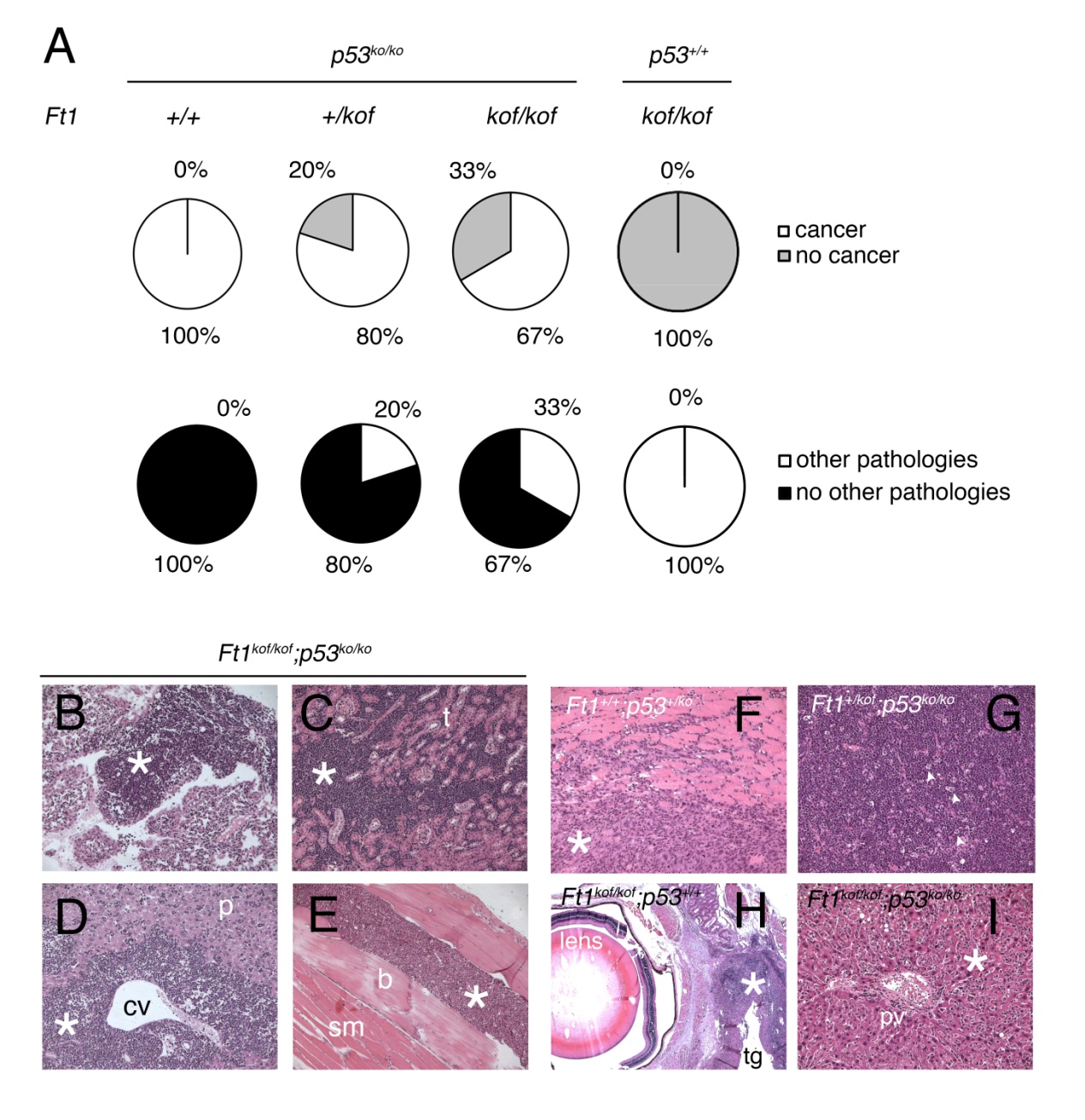
**

**Figure S4**

**
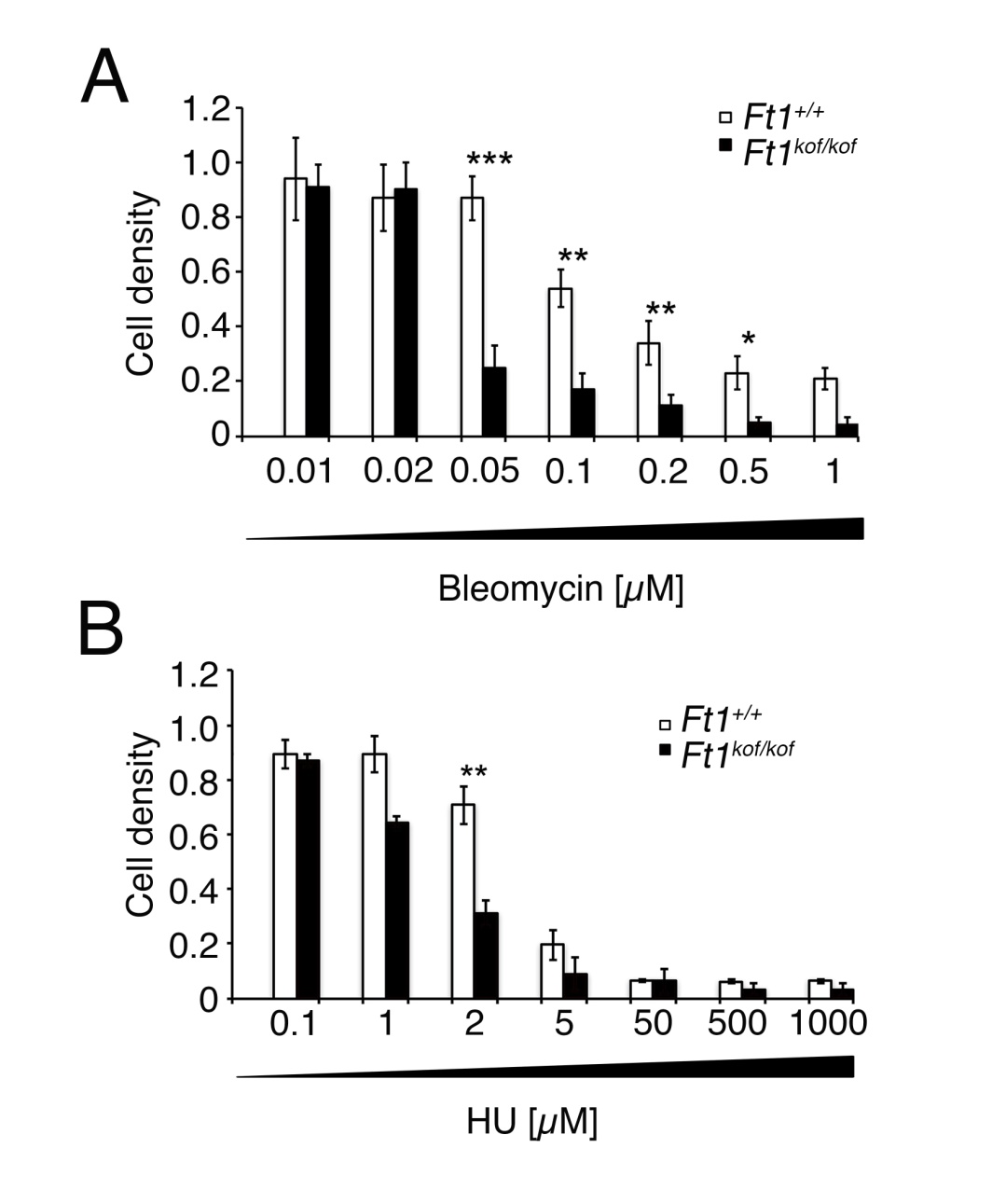
**

**Figure S5**

**
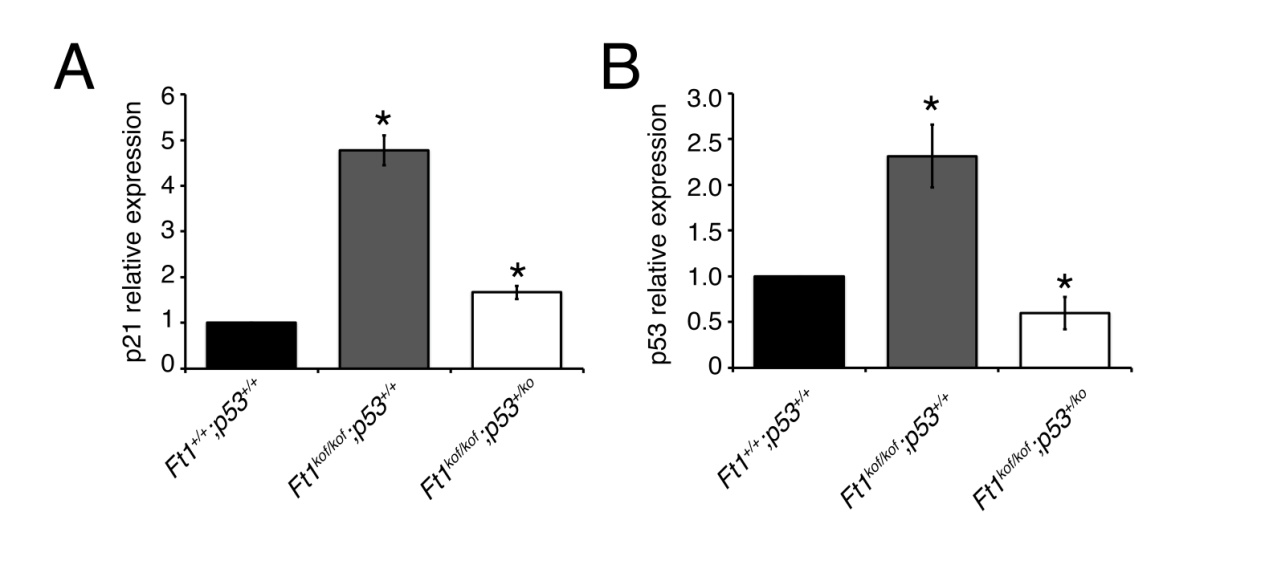
**

**Supplementary figure legend**

**Figure S1, Related to figure 2. Telomere defects observed in *Ft1* mutant MEFs and Ft1-lamin A co-localization in wt MEFs.** (A, B) Percent of signal-free chromosome ends per metaphase (A), and of telomeric fusions per chromosome (B) observed in *Ft1^+/+^* or *Ft1^kof/kof^* MEFs. Graphs show mean ± SEM; differences are not significant in χ2 test. (C) Quantification of TIFs *Ft1^kof/kof^* as compared to wt MEFs. * indicate p<0.05 in the Student t test. (D) Representative images of TIFs in *Ft1^kof/kof^* MEFs. Arrowheads indicate TIFs. Scale bar 5μm. (E) Representative images of MTS, STAs and TFs in *Ft1^kof/kof^* MEFs. (F) Co-immunofluorescence showing partial co-localization of lamin A and Ft1 in wt MEFs. Scale bar 5μm.

**Figure S2, Related to figure 6. *p53* and *Ft1* mutation.** (A) Schematic representation of *Ft1 kof* allele (up) and *p53 ko* (bottom) allele. The insertion of the neomycin gene between exons 6 and 7 disrupts p53 expression. (B) Representative PCR analysis used to detect *Ft1 kof* allele (up) and *p53 ko* allele (bottom) on genomic DNA obtained from pups at weaning. The picture shows different genotypes obtained from *Ft1^+/kof^; p53^+/ko^* intercrosses.

**Figure S3, Related to figure 6. Pathology incidence in mice bearing mutations in *Ft1* and/or p53*.*** (A) Case analysis on animals bearing different combinations of *p53* and/or *Ft1* mutations; note that *Ft1* mutations are mildly cancer protective. For details on single animals see table S1. (B-E) Multiorgan lymphomas observed in *Ft1* and *p53* doubly mutant mice, affecting lung (B), kidney (C), liver (D) and bone (E). Note cancer cells (asterisks); t, renal tubules; p, liver parenchyma; cv, centrilobular vein; b, normal bone; sm, striated muscle. (F-G) Images of local sarcoma (F) and lymphoma (G) in *p53 ko* mice (*Ft1^+/+^; p53^ko/ko^*). *, cancer cells; sm, skeletal muscle; arrowhead, mitotic cells. (H-I) Inflammation in mice bearing mutations in *Ft1* and/or *p53*. (H) Eye chronic phlogosis in *Ft1^kof/kof^* (I) hepatitis in a *Ft1^kof/kof^; p53^ko/ko^* mouse tg, tear gland; *, inflammatory cell aggregate; pv, portal vein. See also Figure S2 and Table S1.

**Figure S4, Related to figure 6. Sensitivity of *Ft1* mutant cells to bleomycin and hydroxyurea.** Cell survival response of MEFs derived from *Ft1^kof/kof^* and wt animals upon increasing doses of bleomycin (A) or hydroxyurea (B), showing that *Ft1^kof/kof^* cells are more sensitive to these drugs than their wt counterparts. Graphs show mean ± SEM; * p<0.05; ** p<0.01; *** p<0.001 in Student’s t test.

**Figure S5, Related to figure 6.** Quantification of Western blotting analysis of p21 and p53 in MEFs from *Ft1^+/+^; p53^+/+^, Ft1^kof/kof^; p53^+/+^* and *Ft1^kof/kof^; p53^+/-^.* Western blotting signals were quantified by Image J software, results are reported relative to expression in wt animals (*Ft1^+/+^;p53^+/+^).* Graphs show mean ± SEM; * p<0.05 in Student’s t test.

**Tables**

| **mice** | **sex** | ***Ft1*** | ***p53*** | **age at death (months)** | **tumor type** | **tumor site** | **other pathologies** |
| --- | --- | --- | --- | --- | --- | --- | --- |
| #136 | ♂ | *+/+* | *ko/ko* | 9 | lymphoma | abdomen |  |
| #243 | ♂ | *+/+* | *ko/ko* | 6 | lymphoma | thymus |  |
|  |  |  |  |  | angiosarcoma | leg |  |
| #188 | ♀ | *+/+* | *+/ko* | 8 | osteosarcoma | leg |  |
| #105 | ♂ | *+/+* | *ko/ko* | 6 | angiosarcoma | leg |  |
| #270 | ♀ | *+/+* | *+/ko* | 12 | squamous carcinoma | skin | hepatitis |
| #187 | ♀ | *+/kof* | *ko/ko* | 6 | lymphoma | spleen |  |
| #242 | ♂ | *+/kof* | *ko/ko* | 4 | lymphoma | multiorgan |  |
| #12 | ♂ | *+/kof* | *+/ko* | 17 | lymphoma | multiorgan |  |
| #114 | ♀ | *+/kof* | *+/ko* | 14 | lymphoma | multiorgan |  |
|  |  |  |  |  | osteosarcoma | leg |  |
| #5 | ♀ | *+/kof* | *+/ko* | 27 | osteosarcoma | uterus |  |
| #75 | ♂ | *+/kof* | *+/ko* | 12 | osteosarcoma | leg |  |
| #177 | ♂ | *+/kof* | *ko/ko* | 8 | angiosarcoma | multiorgan |  |
| #301 | ♂ | *+/kof* | *ko/ko* | 5 | angiosarcoma | rib cage |  |
|  |  |  |  |  | carcinoma | liver |  |
| #60 | ♂ | *+/kof* | *+/ko* | 15 | no tumor |  | nd |
| #241 | ♂ | *+/kof* | *ko/ko* | 4 | no tumor |  | bone marrow aplasia |
| #93 | ♀ | *+/kof* | *+/ko* | 10 | no tumor |  | peritonitis |
| #327 | ♂ | *kof/kof* | *ko/ko* | 8 | lymphoma | multiorgan |  |
| #209 | ♂ | *kof/kof* | *+/ko* | 2 | angiosarcoma | kidney infiltrates liver |  |
| #395 | ♀ | *kof/kof* | *ko/ko* | 8 | rabdomiosarcoma |  | severe hepatitis, nephritis, pneumonia |
| #445 | ♀ | *kof/kof* | *ko/ko* | 6 | no tumor |  | hepatitis |
| #65 | ♂ | *kof/kof* | *+/+* | 1 | no tumor |  | chronic eye infection |
| #195 | ♀ | *kof/kof* | *+/+* | 13 | benign tumor (mesodermic origin) | muscle |  |

**Table S1, Related to figure 6. Case analysis of tumors and other pathologies in mutant mice.** Data were obtained from the histopathological analysis from H&E stained biopsies on animals sacrificed at the occurrence of deadly status.

|  | Age interval (weeks) | n entering the age interval (Nx) | n censored subjects (Cx) | n deaths (Dx) |
| --- | --- | --- | --- | --- |
| *Ft1^+/+^* | 0-136 | 496 | 491 | 5 |
| *Ft1^kof/kof^* | 0-136 | 119 | 102 | 17 |

**Survival Table related to Figure 3D**

|  | Age interval (weeks) | n entering the age interval (Nx) | n censored subjects (Cx) | n deaths (Dx) |
| --- | --- | --- | --- | --- |
| *Ft1^+/+^;p53^+/ko^* | 0-57 | 4 | 0 | 4 |
| *Ft1^+/kof^;p53^+/ko^* | 0-115 | 15 | 0 | 15 |
| *Ft1^kof/kof^;p53^+/ko^* | 0-54 | 7 | 0 | 7 |
| *Ft1^kof/kof^;p53^+/+^* | 0-56 | 3 | 0 | 3 |

**Survival Table related to Figure 6C**
