# Supplementary material for: Mice with reduced expression of the telomere‐associated protein Ft1 develop p53‐sensitive progeroid traits
Source: Aging Cell. 2018 Apr 10;17(4):e12730. doi: 10.1111/acel.12730 (PMC6052474; doi:10.1111/acel.12730)
Supplement: Supplementary file 2 [file ACEL-17-na-s002.docx]

| **Mouse model** | **Lifespan** | **Body weight** | **Skin** | **Heart** | **Fat** | **Skeleton** | **Fertility** | **References** |
| --- | --- | --- | --- | --- | --- | --- | --- | --- |
| *G5/G6-mTerc ko* | Reduced | Reduced | Hair follicles defects | nd | Loss | Kyphosis, bone defects | Infertile | (Lee *et al.* 1998; Rudolph *et al.* 1999; Saeed *et al.* 2011) |
| *K5-TRF1 ko* | Reduced | Reduced | Hair follicles defects | nt | nt | nt | nt | (Martinez *et al.* 2009) |
| *WRN^-/-^ TERC^-/-^* | Reduced | Reduced | Wound healing defects | Failure | Loss | Lordokyphosis, bone defects | nd | (Chang *et al.* 2004) |
| *XPD^R722^* | Reduced | Reduced | Hair follicles defects, skin defects | nc | Loss | Kyphosis, bone defects | Reduced | (de Boer *et al.* 2002) |
| *ERCC1*292 and ERCC ko* | Reduced | Reduced | Skin defects | nc | Reduced | Kyphosis | Reduced | (Weeda *et al.* 1997) |
| *Lmna ^-/-^* | Reduced | Reduced | nd | Aberrant | Reduced | nd | nd | (Sullivan *et al.* 1999) |
| *Lmna^L503P/L503P^* | Reduced | Reduced | Hair follicles defects, skin defects | Aberrant | Loss | Kyphosis, bone defects | nd | (Mounkes *et al.* 2003; Chen *et al.* 2012) |
| *Lmna^G609G/G609G^* | Reduced | Reduced | Hair follicles defects | Aberrant | Loss | Lordokyphosis, bone defects | Infertile | (Osorio *et al.* 2011) |
| *Zmpste24 ko* | Reduced | Reduced | Alopecia | Aberrant | Loss | Kyphosis, bone defects | nd | (Bergo *et al.* 2002; Pendas *et al.* 2002) |
| *Lmna^N195K/N195K^* | Reduced | Reduced | nc | Aberrant | nc | nc | nd | (Mounkes *et al.* 2005) |
| *Ft1^kof/kof^* | Reduced | Reduced | Skin defects | Aberrant | Loss | Lordokyphosis, bone defects | Reduced |  |

nd - not determined; nc - not changed; nt - not targeted.

**Table S2, Related to figures 3-5. Summary table on progeroid models and *Ft1* mutant mice.** Characteristics of progeroid models related to telomeres, DNA damage and lamin-dependent diseases are compared to the phenotypic traits of *Ft1* mutant mice.

**Supplemental references**

Bergo MO, Gavino B, Ross J, Schmidt WK, Hong C, Kendall LV, Mohr A, Meta M, Genant H, Jiang Y, Wisner ER, Van Bruggen N, Carano RA, Michaelis S, Griffey SM, Young SG (2002). Zmpste24 deficiency in mice causes spontaneous bone fractures, muscle weakness, and a prelamin A processing defect. *Proc Natl Acad Sci U S A*. **99**, 13049-13054.

Chang S, Multani AS, Cabrera NG, Naylor ML, Laud P, Lombard D, Pathak S, Guarente L, DePinho RA (2004). Essential role of limiting telomeres in the pathogenesis of Werner syndrome. *Nat Genet*. **36**, 877-882.

Chen CY, Chi YH, Mutalif RA, Starost MF, Myers TG, Anderson SA, Stewart CL, Jeang KT (2012). Accumulation of the inner nuclear envelope protein Sun1 is pathogenic in progeric and dystrophic laminopathies. *Cell*. **149**, 565-577.

de Boer J, Andressoo JO, de Wit J, Huijmans J, Beems RB, van Steeg H, Weeda G, van der Horst GT, van Leeuwen W, Themmen AP, Meradji M, Hoeijmakers JH (2002). Premature aging in mice deficient in DNA repair and transcription. *Science*. **296**, 1276-1279.

Lee HW, Blasco MA, Gottlieb GJ, Horner JW, 2nd, Greider CW, DePinho RA (1998). Essential role of mouse telomerase in highly proliferative organs. *Nature*. **392**, 569-574.

Martinez P, Thanasoula M, Munoz P, Liao C, Tejera A, McNees C, Flores JM, Fernandez-Capetillo O, Tarsounas M, Blasco MA (2009). Increased telomere fragility and fusions resulting from TRF1 deficiency lead to degenerative pathologies and increased cancer in mice. *Genes & development*. **23**, 2060-2075.

Mounkes LC, Kozlov S, Hernandez L, Sullivan T, Stewart CL (2003). A progeroid syndrome in mice is caused by defects in A-type lamins. *Nature*. **423**, 298-301.

Mounkes LC, Kozlov SV, Rottman JN, Stewart CL (2005). Expression of an LMNA-N195K variant of A-type lamins results in cardiac conduction defects and death in mice. *Hum Mol Genet*. **14**, 2167-2180.

Osorio FG, Navarro CL, Cadinanos J, Lopez-Mejia IC, Quiros PM, Bartoli C, Rivera J, Tazi J, Guzman G, Varela I, Depetris D, de Carlos F, Cobo J, Andres V, De Sandre-Giovannoli A, Freije JM, Levy N, Lopez-Otin C (2011). Splicing-directed therapy in a new mouse model of human accelerated aging. *Sci Transl Med*. **3**, 106ra107.

Pendas AM, Zhou Z, Cadinanos J, Freije JM, Wang J, Hultenby K, Astudillo A, Wernerson A, Rodriguez F, Tryggvason K, Lopez-Otin C (2002). Defective prelamin A processing and muscular and adipocyte alterations in Zmpste24 metalloproteinase-deficient mice. *Nat Genet*. **31**, 94-99.

Rudolph KL, Chang S, Lee HW, Blasco M, Gottlieb GJ, Greider C, DePinho RA (1999). Longevity, stress response, and cancer in aging telomerase-deficient mice. *Cell*. **96**, 701-712.

Saeed H, Abdallah BM, Ditzel N, Catala-Lehnen P, Qiu W, Amling M, Kassem M (2011). Telomerase-deficient mice exhibit bone loss owing to defects in osteoblasts and increased osteoclastogenesis by inflammatory microenvironment. *Journal of bone and mineral research : the official journal of the American Society for Bone and Mineral Research*. **26**, 1494-1505.

Sullivan T, Escalante-Alcalde D, Bhatt H, Anver M, Bhat N, Nagashima K, Stewart CL, Burke B (1999). Loss of A-type lamin expression compromises nuclear envelope integrity leading to muscular dystrophy. *J Cell Biol*. **147**, 913-920.

Weeda G, Donker I, de Wit J, Morreau H, Janssens R, Vissers CJ, Nigg A, van Steeg H, Bootsma D, Hoeijmakers JH (1997). Disruption of mouse ERCC1 results in a novel repair syndrome with growth failure, nuclear abnormalities and senescence. *Curr Biol*. **7**, 427-439.
